# A fluorescent sensor-based tripodal-Bodipy for Cu (II) ions: bio-imaging on cells

**DOI:** 10.3906/kim-2107-8

**Published:** 2021-10-21

**Authors:** Ahmed Nuri KURŞUNLU, Mustafa ÖZMEN, Ersin GÜLER

**Affiliations:** Department of Chemistry, University of Selçuk, Konya, Turkey

**Keywords:** Yeast, Bodipy, copper, Uv-vis, fluorescence

## Abstract

A general synthetic method was improved to synthesize a chemosensor based on a tripodal Bodipy (***t*-BODIPY**) structure. The product and its intermediate products were successfully prepared and fully characterized. The metal ion sensing performance of the tripodal compound was evaluated by UV/Vis and fluorescence spectroscopies. According to the obtained data, ***t*-BODIPY** is a selective and sensitive fluorescence probe for detection of Cu^2+^ ions in the presence and in the absence of competing ions. This chemosensor presents relatively a low detection limit of 5.4×10^−7^ M for Cu^2+^. Bio-imaging studies on living yeast cells suggest that ***t*-BODIPY** has some advantages over other chemosensors to recognize copper (II) ions.

## 1. Introduction

Sensitive and selective recognition of metal ions is a crucial problem in many branches of science and engineering. The use of fluorescence sensor systems is undoubtedly the most advantageous technique for metal determination when compared to other conventional analytical techniques [1–3]. Metal ions causing pollution can interact with the sensor ligands through complexation, which would lead to a change in its fluorescence wavelength or intensity [4–6]. The use of Bodipy compounds has been reported in the field of fluorescent probes for the detection of various cations [7–9]. Reportedly, Bodipy is of noteworthy synthetic versatility, high solubility, stability, and optical properties Bodipy can also be used as a ligand for metals, which require different coordination spheres [10–13]. Although these Bodipy fluorescent compounds exhibited a good selectivity&sensitivity for the recognition of metal cations, the reported chemosensors were not soluble in pure water [14–17]. The solubility in aqueous or half-aqueous media is very important to develop biological applications. The number of studies based on Bodipy containing molecules soluble in aqueous or half-aqueous media in the field of sensor science is relatively low [18–23].

With these points in mind, we decided to design a tripodal Bodipy derivative (t-BODIPY) including donor atoms (nitrogen and oxygen) of ligands in order to improve water solubility. It is estimated that the Bodipy structure, which contains multiple donor atoms, can selectively bind to metal cations. Due to their fluorescent sensor properties, ***t-BODIPY*** may find practical applications in the future.

## 2. Experimental methods

Chemicals were supplied by Sigma-Aldrich, Acros and Alfa Aesar. Deuterated solvents were purchased from Merck. Except for solvents, which were degassed with argon for 30 min, all reagents were used without further purification. Nuclear Magnetic Resonance (NMR) spectra (^1^H, ^11^B, ^13^C-NMR and ^19^F) were measured on a Varian (400 MHz) and Bruker (600 Mhz) spectrophotometers at 25 °C. FT-IR measurements were obtained from a Bruker spectrophotometer (Vertex 70/80). The elemental analysis was calculated using a TruSpec analyser. The microscopy (lens: 100×, 50×, 20×, 10×, 4×) images of yeast cells were performed using an Olympus microscope. The cell images were taken by using a CCD camera having million-pixel resolution (DP70 12.5). The images were captured with DP Manager software. FTIC filter was performed for the fluorescence light. The absorption and emission measurements were performed by acetate salts in half-aqueous medium using a Shimadzu 1280 apparatus and PerkinElmer LS 55, respectively. Fresh compressed yeast was purchased from a local grocery store and suspended in an Eppendorf tube with ultrapure water at a concentration of about 30 mg/mL. To purify from impurities, the same processes were repeated 3 times after centrifugation with ultrapure water. The surface of the yeast cells was charged PAH (Poly(allylamine hydrochloride)) (0.5 M NaCl in 10 mg/mL solution) with polyelectrolyte. This process was mixed with 2 mL of PAH/aqueous solution of 300 μL aqueous yeast cell suspension, incubated for 10 min, and the suspension was centrifuged. The supernatant polyelectrolyte was removed, and the yeast cells were washed four times with ultrapure water. Similarly, yeast cells were mixed with ***t*-BODIPY**, which was incubated for 15 min. Then, the suspension was centrifuged. The supernatant free ***t*-BODIPY** was detached, and the yeast cells were washed three times with distilled water. The prepared yeast cells were suspended and examined by optical and fluorescence microscopy.

### 2.1. Synthesis

#### 2.1.1. The preparing of.8-{4-(chloromethyl)phenyl.}-2,6-diethyl-4,4-difluoro-1,3,5,7-tetramethyl-4-bora-3a,4a-diaza-s-indacene (Compound 1)

Compound **1** was synthesized by a known method in our reported previous articles [24]. *4*-(chloromethyl)benzoylchloride (0.01. mol, 1.875 g) was injected using a syringe to the solution of Kryptopyrrole (2.6 mL, 0.02 mol ) in DCM, 200 mL at r. t.. under argon. Then, the final mixture was reacted for 20 min. Following the solution being cooled, triethylamine (2.5 mL) was added to the solution. It was mixed at r.t. for 30 min, and BF_3_.EtO_2_ (2 mL) was finally injected by a syringe. The solution was refluxed in 70 °C for 20 min, and the raw product was purified by a mixture of EtOAc/cyclohexane in 1:8 ratio (yield 39%, 3.45 g). M. P. 188–190 °C. ^1^H NMR [CDCl_3_, 400 MHz]: 7.43 (Ar, d, *J*=7.8, Hz, 2H), 7.21 (ArH, d, *J*=7.8, 2H), 4.64 (2H, CH_2_, s), 2.45 (6H, CH_3_, s) 2.24 (4H, CH_2_, q) 1.28 (6H, CH_3_, s) 0.91 (CH_3_, t, *J*=8.0, 6H). ^13^C NMR [CDCl_3_,100 MHz]: δ (ppm); 153.99, 139.53, 138.87, 135.76, 135.99, 132.98, 130.65, 129.01, 128.54, 45.65, 16.91, 14.65, 12.55, 11.65. Analy. Cal. for (%) C_24_ N_2_H_28_F_2_ClB: C, 67.22; N, 6.53 H, 6.58; Found: C, 66.99; N, 6.12, H, 6.98. MS for C_24_N_2_ H_28_F_2_ClB: 428 [M+H]^+^.

#### 2.1.2. The preparing of (8-{4-(azidomethyl)phenyl}.-2,6-diethyl-4,4-difluoro-1,3,5,7-tetra. methyl-4-bora-3a,4a-diaza-s-indacene) (Compound 2)

NaN_3_ (0.688 g, 1.06 mol) and Compound **1** (0.88 mmol) were stirred in DMF (40 mL) for overnight at r. t. under N_2_ [24]. The mixture was extracted with water/ diethyl ether. The diethyl ether phases were collected and dried with Na_2_SO_4_. Diethyl ether was evaporated, and raw product was obtained using column chromatography (EtOAc/Petroleum ether 1:3) (1.10 g, 94%). M. P.: 139–141 °C. ^1^H-NMR [CDCl_3_, 400 MHz]: 7.46 (2H ArH, d), 7.32 (2H, ArH, d) 4.49 (2H, CH_2_, s), 2.58 (6H, CH_3_, s), 2.33 (4H, CH_2_, q), 1.29 (6H, CH_3_, s), 0.98 (6H, CH_3_, t). ^13^C NMR [CDCl_3_, 100 MHz]: δ (ppm); 11.98. 12.33, 15.03, 17.45, 55.35, 129.04, 128.28, 130.82, 132.94, 135.63, 135.99, 138.04, 138.98, 154.05. Elem. Analy Found: H, 6.68; C, 66.39; N, 16.14, Analytical Cal. for (%) C_24_H_28_BF_2_N_5_: H, 6.48; C, 66.22; N, 16.09.

#### **2.1.3**. The synthesis of (8-{4-(aminomethyl)phenyl}.-2,6-diethyl-4,4.-difluoro-1,3,5,7-tetramethyl.-4-bora-3a,4a-diaza.-s-indacene) (Compound 3)

Compound 2 (0.01 mol, 0.9 g) and TPP (triphenylphosphine) (0.262 2 g, 0.01 mol,) were dissolved in dry THF (150 mL). Following six hours, water (2 drops) was dropped [24]. The solution was mixed for 24 h. The purification of the product was accomplished with a column by a long process (DCM/MeOH 10:1). The yield is calculated as 71% (0.31 g). M.P.≈170 °C.

^1^H-NMR [CDCl_3_, 400 MHz]: 7.45 (2H, ArH, d,), 7.27 (2H, ArH, d), 4.04 (2H, CH_2_, s), 2.57 (6H, CH_3_, s) 2.38 (4H, CH_2_, q) 1.23 (6H, CH_3_, s) 1.03 (6H, CH_3_, t). ^13^C NMR [CDCl_3_, 100 MHz]: δ (ppm); 11.96, 12.55, 14.92, 17.52, 46.32, 127.88, 128.21, 130.98, 133.12, 135.02, 137.02, 140.02, 142.13, 154.36. Analytical Found: H, 7.69; C, 70.56; N, 10.11. Cal. for (%) C_24_H_30_BF_2_N_3_: H, 7.38; C, 70.43; N, 10.28.

#### *2.1.4*. The synthesis of *t-BODIPY*

To a mixture of Compound 3 (1 mmol, 0.41 g,) in dichloromethane, 0.5 mL of diethylisopyropylamine was injected at −4°C (salt./ice). 0.15 g of 1,3,5-benzentricarbonyl chloride was poured to this mixture at r. t. and stirred for 72 h. The solution was extracted in chloroform/water for three times, and the crude residue was drawn into the chloroform. The product was purified using column chromatography (Petroleum Ether/EtOAc; 1:1 after the chloroform was evaporated in vacuo. Dark red solid was obtained. 0.16 g, Yield: 28% (Scheme). ^1^H-NMR [CDCl_3_, 400 MHz]: 8.65 (3H, bs, NH), 8.39 (3H, s, ArH), 7.48–7.15 (12H, m, ArH), 4.48 (CH_2_, s, 6H,), 2.48 (CH_3_, s, 18H,), 2.15 (CH_2_, q, 12H), 1.60 (CH_3_, s, 18H), 1.12 (CH_3_, t, 12H). ^13^C-NMR [CDCl_3_, 100 MHz]: 167.8, 148.3, 145.4, 140.2, 138.5, 137.2, 135.1, 130.1, 129.7, 125.3, 123.7, 118.3, 114.2, 43.1, 18.2, 17.2, 15.1, 14.1, 12.2, 11.3. Analy. Calcl. (%) C_81_H_90_B_3_F_6_N_9_O_3_; C, 70.29; H,. 6.55; N, 9.11 Found; C, 70.52; H, 6.77; N, 9.01. MS [+H^+^]; m/z: 1385.1

## 3. Results and discussion

In the absorption study for the chemosensor, the solution of the ligand was prepared at concentrations of 1.10^−6^ M in methanol, and counter ions (Mn (II), Cr (III), Fe (II), Li (I), Co (II), Zn (II), Al (III), Ni (II), Ga (III), Cd (II), Cu (II), Ag (I), Hg (II)) solutions were prepared in water at 20.10^−6^ M concentration. The absorption, emission, and the related spectroscopic calculations were made by mixings of metal salts and ***t*-BODIPY** solutions in a 1:1 ratio (v:v).

When FT-IR spectra of compounds (Figures S1–S4) was examined, peaks around 2800–3000 cm^−1^ were assigned to aromatic or aliphatic C-H vibrations and the peaks around 1680 cm^−1^ are attributed to C=O vibrations. Moreover, C=C and C=N vibration peaks were observed among 1600–1400 cm^−1^ as multisignals and 1640 cm^−1^, respectively.

Four main transitions were observed in the absorption spectrum of ***t*-BODIPY** where these bands appeared around 230, 270, 370, and 530 nm, respectively. The molar absorption coefficient was calculated as 161000 M^−1^.cm^−1^ due to behaviors of different auxochrome moieties involving the lone pair of electrons while the absorption maximum is not shifted.

**Figure 1:**
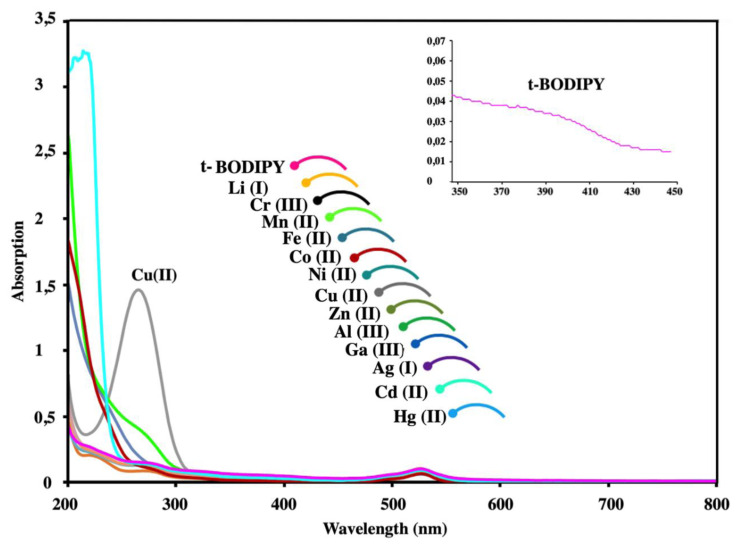
Absorption spectral changes of ***t-*BODIPY** upon addition of various metal ions ((Cr (III), Li (I), Fe (II), Mn (II), Ni (II), Co (II), Zn (II), Cu (II), Al (III), Cd (II), Ga (III), Ag (I), Hg (II)).

The band around 370 nm indicates that the π-π * transitions assigned to the aromatic groups of the chemosensor ***t*-BODIPY** in a three-way molecular structure (Figure 1a). Also, another sharp band observed at 530 nm indicates the classical S_0_-S transition of Bodipy compounds. In contrast to minor changes after the interaction between most metal ions with ***t*-BODIPY** solutions, Cu (II) ions lead to a significant change in the absorption band around 280 nm. This rising band may be associated with the formation of a complex. Since ***t*-BODIPY**'s absorption spectrum showed a significant change only in the presence of Cu (II) ions, ***t*-BODIPY** might be used to detect Cu (II) ions. It is thought that the Cu (II) ion interacts with the nitrogen and oxygen atoms of the amide parts of ***t*-BODIPY** to form a complex (Figure 1b and S18). The aromatic region peaks slightly shifted to a different ppm value while NH broad peak completely disappeared and compared (Figure S19,S20). NMR spectrum of the complex form between ***t*-BODIPY** and Cu (II) was also supported with both Job plot and previous literature [25].

**Figure 2:**
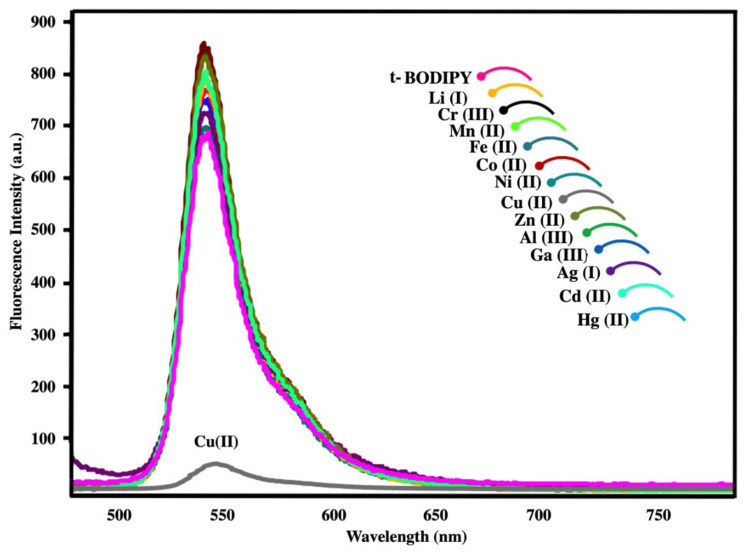
Fluorescence spectral changes of ***t-BODIPY*** upon addition of various metal ions (Cr (III), Li (I), Fe (II), Mn (II), Ni (II), Co(II), Zn (II), Cu (II), Al (III), Cd (II), Ga (III), Ag (I), Hg (II)); λex:470 nm.

To support absorption results, the emission spectra of the ***t*-BODIPY**-metal ion mixtures were also investigated (Figure 2). The fluorescence spectra of the prepared ***t*-BODIPY** and ***t*-BODIPY**-metal ion mixtures were excited at 470 nm to an optimum result. As shown in

Figure 2, *t***-BODIPY** emission band at 545 nm has changed only in the presence of copper (II) ion just like in the absorption studies. Here, the fluorescence intensity of ***t*-BODIPY** has changed significantly, quenched (over 90%) towards 60 units from a fluorescence intensity of 700 units. This effect can likewise be attributed to the complex interaction between nitrogen and oxygen atoms in the amide groups and copper (II) ions. Moreover, the emission intensity of ***t*-BODIPY** did not almost change in the presence of other copper salts (F^−^, Cl^−^, I^−^, Br^−^, HCO_3_^−^, CO_3_^2−^, HSO_3_^−^, SO_4_^2−^, NO_3_^−^) (Figure S21).

**Figure 3:**
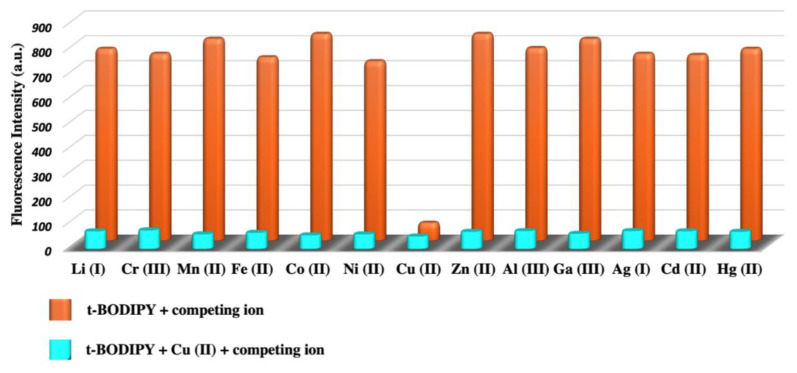
Fluorescence intensities around 548 nm of ***t-*BODIPY**&Cu (II) ion mixture in the presence of a series of metal ions (Cr(III), Li (I), Fe (II), Mn (II), Ni (II), Co (II), Zn (II), Cu (II), Al(III), Cd (II), Ga (III), Ag (I), Hg (II)).

Another selectivity study of ***t*-BODIPY**, where copper (II) ion sensitivity was also confirmed, was conducted. For this aim, ***t*-BODIPY**+ Cu (II) + other ion solutions were prepared. Other studied metal ions did not change the selectivity of the copper (II), and the intensity of the quenched fluorescence of ***t*-BODIPY** did not change (Figure 3).

**Figure 4:**
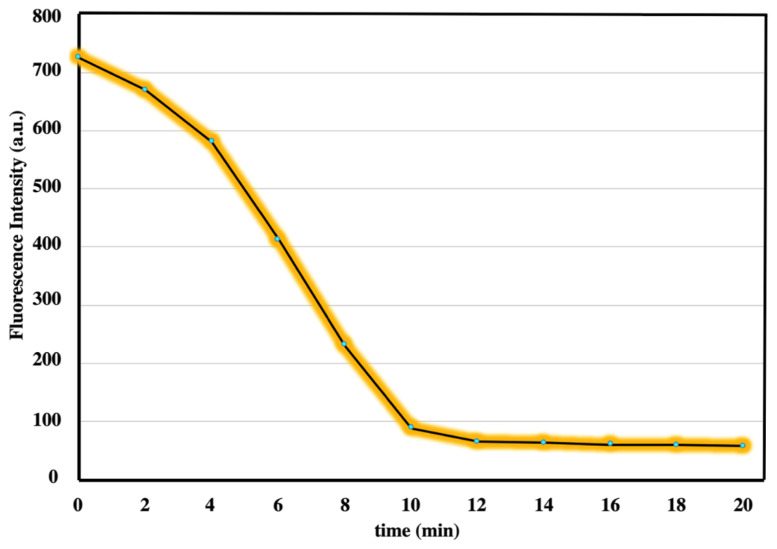
Response time experiments based-on the complexation between Cu (II) and ***t*-BODIPY** in the half-aqueous medium(λem_max_ = 547 nm).

Emission measurements were taken at 1, 2, 3, 4, 5, 10, 15, and 20 min after adding of metal ion for the best response time for ***t*-BODIPY**, which has a sensor feature for copper ions (Figure 4). As it can be understood from Figure 4, *t***-BODIPY**'s fluorescence intensity decreased continuously, and, after 10 min, this effect almost ended. The ideal response time of this ***t*-BODIPY** was determined as ten minutes at room temperature.

**Figure 5:**
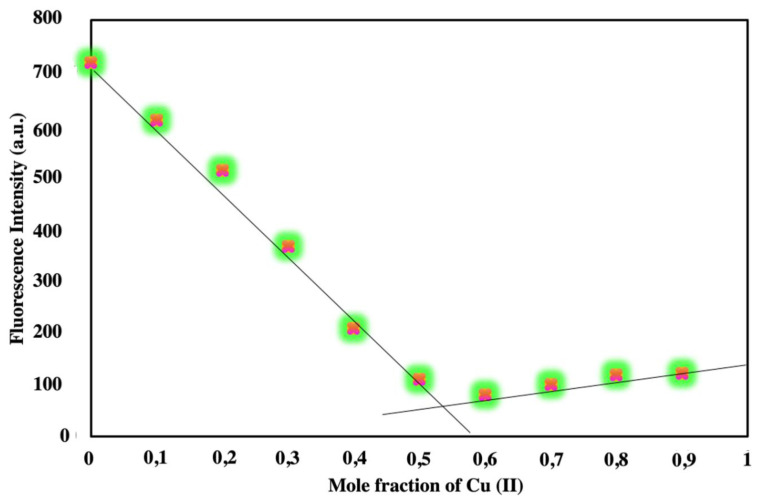
Job’s plot graph of the complex carried out for ***t*-BODIPY** with Cu (II).

The stoichiometry of the complexometric interaction between Cu (II) ion and ***t*-BODIPY** was determined using Job’s method (Figure 5). As can be seen from Figure 5, the minimum emission is located at the center, at the point corresponding to the 0.5 mole fraction with 1/1 metal/ligand ratio. Due to the N atom in the amide group being a hydrogen bond donor, it plays a vital position in the complex reaction.

**Figure 6:**
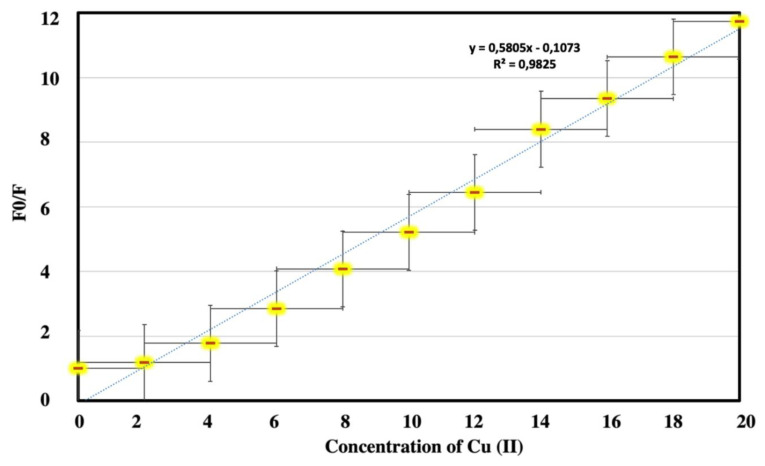
The emission intensities ratio (F_0_/F) around 547 nm plotted against copper (II) ion concentrations (1.10^−6^–2.5×10^−5^ M)(λem_max_ = 548 nm).

Stern-Volmer equation was used for the calculation of the binding constant in the complex. For this aim, the emission maxima of Cu (II)/***t*-BODIPY** solutions were obtained (Figure 6).


$$F_{0}/F=1+K_{\mathrm{sv}}\;[C]$$

Where K; is the binding constant, F_0_; Emission of ***t*-BODIPY** in the absence of copper (II), F; fluorescence intensity in the presence of Cu (II) ion, C; the concentration of Cu (II) ion. As can be seen from Figure 6, the F_0_/F ratio at different concentrations seemed linear and increasing curve. The binding constant (Ksv) was calculated from this equation as 1.68×10^5^ M^−1^.

The limit of detection (LOD) was performed from some parameters (the stand. dev. blank) that affected the accuracy of the model performed to determine concentrations from the fluorescence intensities.


LOD=3s/F

In there, s is the stand. dev. of the blank mixture, F is the false of the LOD curve. The limit of detection (LOD) of copper ion was calculated as 5.4×10^−7^ M by using the fluorescence intensities of ***t*-BODIPY** in Figure 6. When the result is compared with our previous paper concluding yeast cell studies, it can be evaluated as a worse value [29]. However, this LOD value can be accepted based on the U.S.’s defined contaminant level in tap water, which is 20 μM, for copper ions; hence, the probe, ***t*-BODIPY**, is effective for the recognition of copper (II) in real samples. ***t*-BODIPY** has a lower LOD that is beneficial for the recognition of copper (II) in the half-aqueous medium when compared with previous literature, [15,21,26,27]. The selectivity performance of ***t*-BODIPY** towards copper (II) was also compared with some published probes for copper (II) through a complex mechanism. As understood from Table, ***t*-BODIPY** can challenge the detection of copper ions toward some Bodipy-based fluorescent chemosensors operated with turn-off&PET mechanism [15,21,26,27]. The emission intense of ***t*-BODIPY** is quenched by the amide fragments on triple Bodipy units. ***t*-BODIPY** has many attractive compensations in terms of detection limit, sensing techniques, and yeast living-cells.

**Figure 7:**
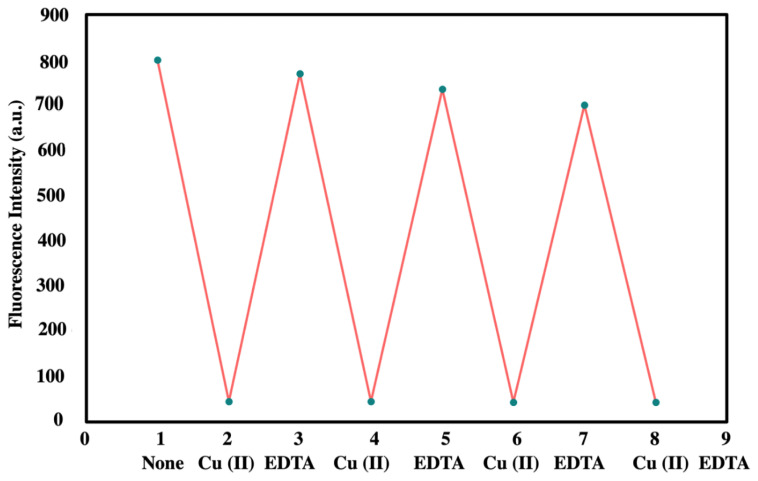
The sequential recognition of ***t*-BODIPY** (1.10^−6^ M) upon alternate addition of Cu(II) and EDTA (20.10^−6^ M) inmethanol/H_2_O (9:1 v/v) system (λ_ex_:470).

The stability of ***t*-BODIPY**-Cu (II) was also determined with Cu (II) and EDTA solutions. The solution of Cu (II) (20.10^−6^ M) was firstly added to the mixture of ***t*-BODIPY** (1.10^−6^ M) and ligand’s fluorescence intensity quenched. Then, EDTA (20.10^−6^ M) was dropped to this mixture, and fluorescence intensity reincreased quickly toward 800 units (Figure 7). As shown in Figure 7, the fluorescence intensity of ***t*-BODIPY** quenched in the presence of copper (II) continued to be stable after four times. The recovery rate of the fluorescence intensity following the first adding of EDTA was above 85%, and the three serial recovery rates were slightly reduced.

**Figure 8:**
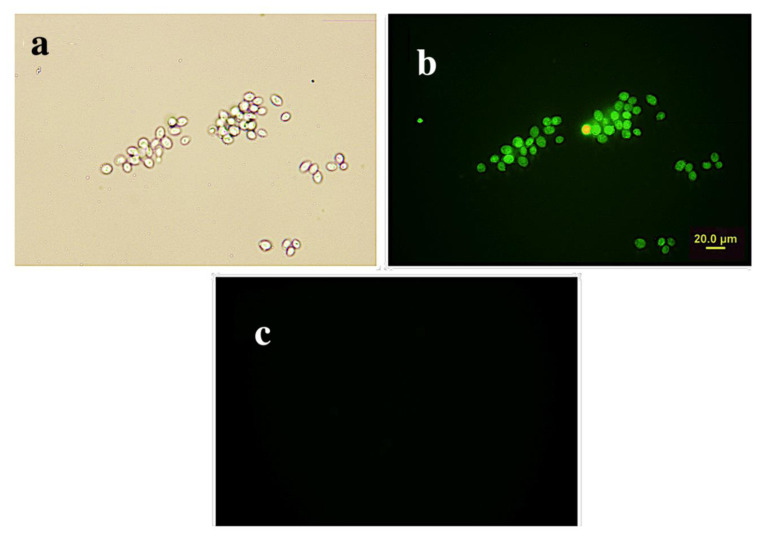
Optical microscope photo of yeast cells (a) and fluorescence microscope image (b) of yeast cells after coupling with ***t*-BODIPY** fluorescence microscopy image after adding Cu (II) solution to Bodily yeast cells (c).

The yeast cells photographed with bright-field optical microscopy (Figures 8a, 8b, 8c). The yeast cells were interacted with ***t*-BODIPY** and investigated under a fluorescence microscope after the polyelectrolyte coating. As it can be observed from Figure 8b, *t***-BODIPY** interacted with the yeast cells, and then Figure 8c supported that copper (II) ion quenched the emission intensity of ***t*-BODIPY**.

## 4. Conclusion

In summary, a known tripodal fluorescence chemosensor (***t*-BODIPY**) was prepared by the proposed synthetic strategy and showed sensitivity and selectivity for copper (II) ion. The emission and absorption values of ***t*-BODIPY** only changed in the presence of copper(II) ion, and other ions did not cause any change. In the presence of copper (II) ion, the emission of ***t*-BODIPY** showed a significant damping effect without causing any wavelength shift, and the fluorescence intensity decreased. This situation has revealed an effective energy mechanism, and the high sensitivity of ***t*-BODIPY** to Cu (II) ions has been demonstrated. The competing ion study was performed for ***t*-BODIPY**, which has been proven to be sensitive to copper ions and did not cause any significant changes to the ***t*-BODIPY**/Cu (II) complex. ***t*-BODIPY** can be used as a selective&sensitive probe for the copper (II) ion; LOD was determined as 5.4×10^−7^ M. ***t*-BODIPY** was effectively applied in the bio-imaging of copper (II) in yeast cells.

## Experimental Section

### Materials, instruments, and methods

Chemicals were supplied by Sigma-Aldrich, Acros and Alfa Aesar. Deuterated solvents were purchased from Merck. Except for solvents, which were degassed with argon for 30 min, all reagents were used without further purification. Nuclear Magnetic Resonance (NMR) spectra (^1^H, ^11^B, ^19^F, and ^13^C-NMR) were measured on a Varian 400 MHz and Bruker 600 Mhz spectrophotometers at 25 °C. FT-IR measurements were measured using a Bruker (Vertex 70) spectrophotometer, and elemental analysis was performed using a TruSpec analyser. The microscopy (lens: 100×, 50×, 20×, 10×, 4×) images of yeast cells were performed using an Olympus microscope. The images were taken by using a CCD camera (DP70 12.5 million-pixel resolution). The images were captured with DP Manager software. FTIC filter was used for the fluorescence light. The absorption and emission data were performed by metal acetate in a half aqueous medium using a Shimadzu 1280 apparatus and PerkinElmer LS 55, respectively. Fresh compressed yeast was purchased from a local grocery store and suspended in an Eppendorf tube with ultrapure water at a concentration of about 30 mg/mL. To purify from impurities, the same processes were repeated 3 times after centrifugation with ultrapure water. The surface of the yeast cells was charged PAH (Poly(allylamine hydrochloride)) (0.5 M NaCl in 10 mg/mL solution) with polyelectrolyte. This process was mixed with 2 mL of PAH/aqueous solution of 300 μL aqueous yeast cell suspension, incubated for 10 min and then centrifuged. The supernatant polyelectrolyte was removed, and the yeast cells were washed four times with ultrapure water. Similarly, yeast cells were mixed with ***t*-BODIPY** and incubated for 15 min and then centrifuged. The supernatant free ***t*-BODIPY** was removed, and the cells were washed three times with distilled water. The prepared yeast cells were suspended and examined by optical and fluorescence microscopy.

## The synthesis of compounds

### The synthesis of Compound 1 (8-{4-(chloromethyl)phenyl}-2,6-diethyl-4,4-difluoro-1,3,5,7-tetramethyl-4-bora-3a,4a-diaza-s-indacene)

Compound **1** was synthesized by a known method in our reported previous articles [24]. *p*-(chloromethyl)benzoyl chloride (1.875 g, 0.01 mol) was injected by a syringe to the solution of Kryptopyrrole (2.6 mL, 0.02 mol ) in DCM, 200 mL under argon atmosphere at r. t. Then, the mixture was stirred for 20 min. Following the solution was cooled, 2.5 mL of triethylamine was added to the solution, it was mixed at r.t. for 30 min, and boron trifluoride diethyl etherate (2 mL) was finally added by syringe. The mixture was refluxed at 70 °C for 20 min, and the residue was purified by a solution of cyclohexane/ethylacetate in 8:1 ratio (3.47 g, yield 40%). M. P. 189 °C. ^1^H NMR [400 MHz, CDCl_3_]: 7.43 (PhH, d, *J*=7.8, Hz, 2H), 7.21 (PhH, d, *J*=7.8, 2H), 4.64 (CH_2_, s, 2H), 2.45 (CH_3_, s, 6H) 2.24 (CH_2_, q, 4H) 1.28 (CH_3_, s, 6H) 0.91 (CH_3_, t, *J*=8.0, 6H). ^13^C NMR [100 MHz, CDCl_3_]: δ (ppm); 154.03, 139.49, 138.66, 135.88, 136.11, 133.02, 130.77, 128.98, 128.39, 45.65, 16.91, 14.65, 12.55, 11.65. Analytical Cal. for (%) C_24_H_28_N_2_F_2_ClB: H, 6.58; C, 67.22; N, 6.53; Found: H, 6.98; C, 66.99; N, 6.12. MS for C_24_H_28_N_2_F_2_ClB: 428 [M+H]^+^.

### The synthesis of Compound 2 (8-{4-(azidomethyl)phenyl}-2,6-diethyl-4,4-difluoro-1,3,5,7-tetra methyl-4-bora-3a,4a-diaza-s-indacene)

NaN_3_ (0.688 g, 1.06 mol) and Compound **1** (0.88 mmol) were stirred in N,N-dimethylformamide (40 mL) for overnight at room temperature under argon atmosphere [24]. The mixture was extracted with diethyl ether/water. The diethyl ether phases were collected, dried with Na_2_SO_4_. Diethyl ether was evaporated, and raw product was purified in column (ethylacetate/cyclohexane 1:3) (1.10 g, 95%). M. P.: 140 °C. ^1^H-NMR [400 MHz, CDCl_3_]: 7.46 (PhH, d, 2H), 7.32 (PhH, d, 2H,) 4.49 (CH_2_, s, 2H,), 2.58 (CH_3_, s, 6H), 2.33 (CH_2_, q, 4H), 1.29 (CH_3_, s, 6H,), 0.98 (CH_3_, t, 6H). ^13^C NMR [100 MHz, CDCl_3_]: δ (ppm); 154.05, 138.98, 138.04, 135.99, 135.63, 132.94, 130.82, 128.28, 129.04, 55.35, 17.45, 15.03, 12.33, 11.98. Analytical Cal. for (%) C_24_H_28_BF_2_N_5_: H, 6.48; C, 66.22; N, 16.09; Found: H, 6.68; C, 66.39; N, 16.14.

### The synthesis of Compound 3 (8-{4-(aminomethyl)phenyl}-2,6-diethyl-4,4-difluoro-1,3,5,7-tetramethyl-4-bora-3a,4a-diaza-s-indacene)

Triphenylphosphine (0.01 mol, 0.262 2 g) and Compound 2 (0.01 mol, 0.9 g) were dissolved in dry THF (150 mL). Following six hours, 2 drops of distilled water were dropped [24]. The mixture was stirred for 24 h. The purification of the product was accomplished by a long/difficult procedure in column (dichloromethane/methanol 10:1). The yield is calculated as 71% (0.31 g). M.P.≈170 °C.

^1^H-NMR [400 MHz, CDCl_3_]: 7.45 (PhH, d, 2H), 7.27 (PhH, d, 2H) 4.04 (CH_2_, s, 2H), 2.57 (CH_3_, s, 6H) 2.38 (CH_2_, q, 4H,) 1.23 (CH_3_, s, 6H) 1.03 (CH_3_, t, 6H). ^13^C NMR [100 MHz, CDCl_3_]: δ (ppm); 154.36, 142.13, 140.02, 137.02, 135.02, 133.12, 130.98, 128.21, 127.88, 46.32, 17.52, 14.92, 12.55, 11.96. Analytical Cal. for (%) C_24_H_30_BF_2_N_3_: H, 7.38; C, 70.43; N, 10.28; Found: H, 7.68; C, 70.55; N, 10.11.

## The synthesis of t-BODIPY

To a solution of Compound 3 (0.41 g, 1 mmol) in dichloromethane, 0.5 mL of diethylisopyropylamine was injected at −4 °C (salt-ice). Then, 0.15 g of 1,3,5-benzentricarbonyl chloride was poured to this solution at r. t. and stirred for 72 h. The mixture was extracted in water/chloroform for three times and the crude residue was drawn into the chloroform. The chloroform was evaporated in vacuo and the product was purified in the column (cyclohexane/ethylacetate; 1:1). Dark red solid was obtained. 0.16 g, Yield: 28% (Scheme 1). ^1^H-NMR [400 MHz, CDCl_3_]: 8.65 (bs, 3H, NH), 8.39 (s, 3H, ArH), 7.48–7.15 (m, 12H, ArH), 4.48 (s, 6H, CH_2_), 2.48 (s, 18H, CH_3_), 2.15 (q, 12H, CH_2_), 1.60 (s, 18H, CH_3_), 1.12 (t, 12H, CH_3_). ^13^C-NMR [100 MHz CDCl_3_]: 167.8, 148.3, 145.4, 140.2, 138.5, 137.2, 135.1, 130.1, 129.7, 125.3, 123.7, 118.3, 114.2, 43.1, 18.2, 17.2, 15.1, 14.1, 12.2, 11.3. Analy. Calcl. (%) C_81_H_90_B_3_F_6_N_9_O_3_; C, 70.29; H, 6.55; N, 9.11 Found; C, 70.52; H, 6.77; N, 9.01.

Figure S1 FT-IR spectrum of Compound **1**.

Figure S2 FT-IR spectrum of Compound **2**.

Figure S3 FT-IR spectrum of Compound **3**.

Figure S4 FT-IR spectrum of ***t-BODIPY***.

Figure S5 ^1^H-NMR spectrum of Compound **1**.

Figure S6 ^1^H-NMR spectrum of Compound **2 (25 °C)**.

Figure S7 ^1^H-NMR spectrum of Compound **3 (25 °C)**.

Figure S8 ^1^H-NMR spectrum of ***t-BODIPY* (25 °C)**.

Figure S9 ^13^C-NMR spectrum of Compound **2 (25 °C)**.

Figure S10 ^13^C-NMR spectrum of Compound **3 (25 °C)**.

Figure S11 ^13^C-NMR spectrum of ***t-BODIPY* (25 °C)**.

Figure S12 ^11^B-NMR spectrum of Compound **2 (25 °C)**.

Figure S13 **^11^B-NMR** spectrum of Compound **3 (25 °C)**.

Figure S14 ^19^F-NMR spectrum of Compound **2 (25 °C)**.

Figure S15 **^19^F-NMR** spectrum of Compound **3 (25 °C)**.

Figure S16 Mass spectrum of Compound **1**.

Figure S17 Mass spectrum of Compound **3**.

Figure S18 ^1^H NMR titration spectra of ***t-BODIPY*** upon addition of 1 equiv. Cu (II) in DMSO-d6 solution **(25 °C)**.

Figure S19 Mass spectrum of ***t-BODIPY***.

Figure S20 Comparation of ^1^H NMR titration spectra of ***t-BODIPY*** and ***t-BODIPY***-Cu (II) complex.

Figure S21 Fluorescence spectral changes of ***t-BODIPY*** upon addition of various anions (F^−^, Cl^−^, I^−^, Br^−^, CH_3_COO^−^, HCO_3_^−^, CO_3_^2−^, HSO_3_^−^, SO_4_^2−^, NO_3_^−^). λex:470 nm.

## Figures and Tables

**Scheme:**
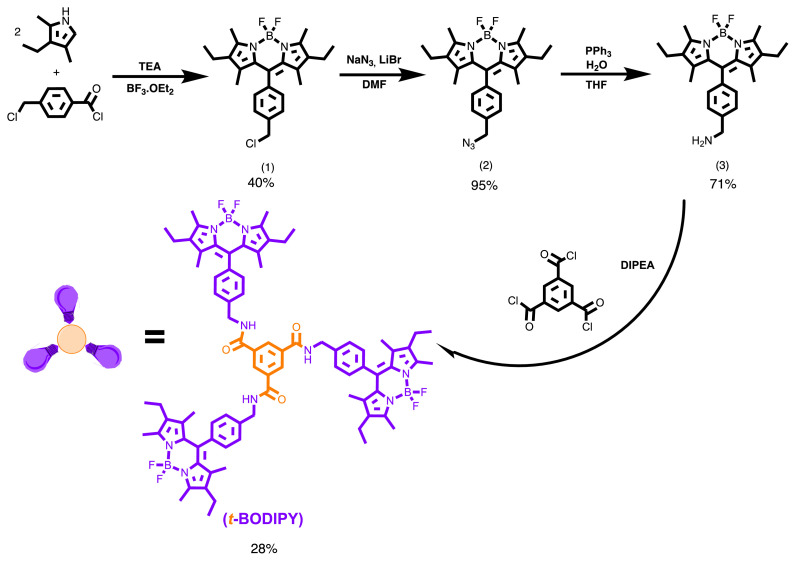
The obtaining route of ***t*-BODIPY**.

**Table:** The comparison of Bodipy chemosensors for Cu (II) with our study.

Mechanism Type	Limit of detection (M)	Ref.
Turn-off & PET	0.11× 10^−6^	[26]
Turn-off & PET	3.97 × 10^−6^	[15]
Turn-off & PET	5.36 × 10^−6^	[15]
Turn-off & PET	0.124 × 10^−6^	[21]
Turn-off & PET-aggregation	-	[28]
Turn-off & PET	3.6 10^−7^	[29]
Turn-off & PET	5.4×10^−7^	Our study

## References

[1] He X-P Song Z Wang Z-Z Shi X-X Shi CK Creation of 3,4-bis-triazolocoumarinesugar conjugates via flourogenic dual click chemistry and their quenching specificity with silver(I) in aqueous media Tetrahedron 2011 67 3343 3347

[2] Raju V Kumar RS Tharakeswar Y Kumar SKA A multifunctional Schiff-base as chromogenic chemosensor for Mn2+ and fluorescent chemosensor for Zn2+ in semi-aqueous environment Inorganic Chimica Acta 2019 493 49 56

[3] Normaya E Fazli M Ahmad MN Bulat KHK COSMO-RS and DFT studies on development and optimization of quercetin as a chemosensor for Fe3+ recognition in aqueous medium Journal of Molecular Structure 2019 1184 538 545

[4] Baruah M Qin W Flors C Hofkens JRA Vallée L Solvent and pH dependent fluorescent properties of a dimethylaminostyryl borondipyrromethene dye in solution Journal of Physical Chemistry A 2006 110 5998 6009 16671668 10.1021/jp054878u

[5] Sinan B A simple rhodanine-based fluorescent sensor for mercury and copper: The recognition of Hg2+ in aqueous solution, and Hg2+/Cu2+ in organic solvent Journal of Photochemistry Photobiology A: Chemistry 2019 372 235 244

[6] Coskun A Akkaya EU Signal Ratio Amplification via Modulation of Resonance Energy Transfer: Proof of Principle in a Novel Emission Ratiometric Hg (II) Sensor Journal of American Chemistry Society 2006 128 14474 14475 10.1021/ja066144g 17090027

[7] Hu B Su Q Lu P Wang YG “BODIPY modified 9-cycloheptatrienylidene fluorene derivatives: Fluorescent "turn-on" for detecting Cu2+ with acidity independence” Sensors Actuator B 2012 168 310 317

[8] Liu C Xiao T Wang Y Wang F Chen X Rhodamine based turn-on fluorescent sensor for Hg2+ and its application of microfluidic system and bioimaging Tetrahedron 2017 73 5189 5193

[9] Li JS Wang H Huang KJ Zhang HS Determination of biogenic amines in apples and wine with 8-phenyl-(4-oxy-acetic acid N-hydroxysuccinimide ester)-4, 4-difluoro-1,3,5,7-tetramethyl-4-bora-3a,4a-diaza-s-indacene by high performance liquid chromatography Analytical Chimica Acta 2006 575 255 261 10.1016/j.aca.2006.05.088 17723599

[10] Kursunlu AN Guler E Ucan HI Boyle RW A novel Bodipy-Dipyrrin fluorescent probe: Synthesis and recognition behaviour towards Fe (II) and Zn (II) Dyes Pigments 2012 94 496 502

[11] Taner B Kursunlu AN Güler E The example of calix[4]pyrrole derivative containing Bodipy unit: Fluorometric and colorimetric sensor for F– ion Spectrochimica Acta Part A: Molecular and Biomolecular Spectroscopy 2014 118 903 907 24161854 10.1016/j.saa.2013.09.077

[12] Quan L Sun T Lin W Guan X Zheng M BODIPY Fluorescent Chemosensor for Cu2+ Detection and Its Applications in Living Cells: Fast Response and High Sensitivity Journal of Fluorescence 2014 24 841 846 24522344 10.1007/s10895-014-1360-9

[13] Kursunlu AN Deveci P Guler E Synthesis and spectroscopic–electrochemical properties of novel ratiometric Hg (II) chemosensor containing Bodipy and the N-phenylaza-15-crown-5 moiety Journal of Luminescence 2013 136 430 436 24496245 10.1016/j.jlumin.2012.12.020 PMC3564662

[14] Yildiz EA Sevinc G Yaglioglu HG Hayvali M Strategies towards enhancing the efficiency of BODIPY dyes in dye sensitized solar cells Journal of Photochemistry Photobiology A: Chemistry 2019 375 148 157

[15] Chen Y Zhao L Jiang J The naphthoate-modifying Cu2+ detective Bodipy sensors with the fluorescent ON-OFF performance unaffected by molecular configuration Spectrochimica Acta Part A: Molecular and Biomolecular Spectroscopy 2017 175 269 275 28064068 10.1016/j.saa.2016.12.034

[16] Goud TV Tutar A Biellmann JF Synthesis of 8-heteroatom-substituted 4,4-difluoro-4-bora-3a,4a-diaza-s-indacene dyes (BODIPY) Tetrahedron 62 2006 5084 5091

[17] Ziessel R Ulrich G Harriman A The chemistry of Bodipy: A new El Dorado for fluorescence tools New Journal Chemistry 2007 31 496 501

[18] Alamiry MAH Benniston AC Copley G Elliott KJ Harriman A A molecular rotor based on an unhindered boron dipyrromethane (Bodipy) dye Chemical Materials 2008 20 4024 4032

[19] Burghart A Kim H Welch MB Thoresen LH Reibenspies J 3,5-diaryl-4,4-difluoro-4-bora-3a,4a-diaza-s-indacene (BODIPY) dyes: Synthesis, spectroscopic, electrochemical, and structural properties Journal of Organic Chemistry 1999 64 7813 7819

[20] Kursunlu AN Şahin E Güler E Bodipy/dipyridylamino-based “turn-on” fluorescent chemosensor for trivalent chromium cations: characterization and photophysical properties RSC Advances 2015 5 5951 5957

[21] Tümay SO Okutan E Sengul IF Özcan E Kandemir H Naked-eye fluorescent sensor for Cu(II) based on indole conjugate BODIPY dye Polyhedron 2016 117 161 171

[22] Şenkuytu E Eçik ET Çoşut B Bodipy decorated triazine chemosensors for Ag+ ions with high selectivity and sensitivity Journal of Luminescence 2018 203 639 645

[23] Xue Z Liu T Liu H Naked-eye chromogenic and fluorogenic chemosensor for mercury (II) ion based on substituted distyryl BODIPY complex Dyes and Pigments 2019 165 65 70

[24] Baslak C Kursunlu AN A naked-eye fluorescent sensor for copper (II) ions based on a naphthalene conjugate Bodipy dye Photochemical & Photobiological Sciences 2018 17 1091 1097 29947409 10.1039/c8pp00137e

[25] He S-J Xie Y-W Chen Q-Y A NIR-BODIPY derivative for sensing copper (II) in blood and mitochondrial imaging Spectrochimica Acta Part A: Molecular and Biomolecular Spectroscopy 2018 195 210 214 29414580 10.1016/j.saa.2018.01.076

[26] Chen Y Pan H Wang F Zhao Y Yin H An ultrafast BODIPY single molecular sensor for multi-analytes (acid/base/Cu2+/Bi3+) with different sensing mechanism Dyes and Pigments 2019 165 279 286

[27] Kursunlu AN Ozmen Mustafa Güler Ersin A Novel Fluorescent Chemosensor for Cu (II) Ion: Click Synthesis of Dual-Bodipy Including the Triazole Groups and Bioimaging of Yeast Cells Journal of Fluorescence 2019 29 1321 1329 31713767 10.1007/s10895-019-02456-3

[28] Zhang Y-M Zhu W Zhao Q Qu W-J Yao H Th4+ tuned aggregation-induced emission: A novel strategy forsequential ultrasensitive detection and separation of Th4+ and Hg2+ Spectrochimica Acta Part A: Molecular and Biomolecular Spectroscopy 2020 229 117926 31855813 10.1016/j.saa.2019.117926

[29] Li S Cao D Hu Z Li Z Meng X A chemosensor with a paddle structure based on a BODIPY chromophore for sequential recognition of Cu^2+^ and HSO_3_^−^ RSC Advances 2019 9 34652 34657 35530010 10.1039/c9ra08345f PMC9073911

