# Supplementary figures and images for: A fluorescent sensor-based tripodal-Bodipy for Cu (II) ions: bio-imaging on cells
Source: Turk J Chem. 2021 Oct 21;45(6):2024–33. doi: 10.3906/kim-2107-8 (PMC10734719; doi:10.3906/kim-2107-8)

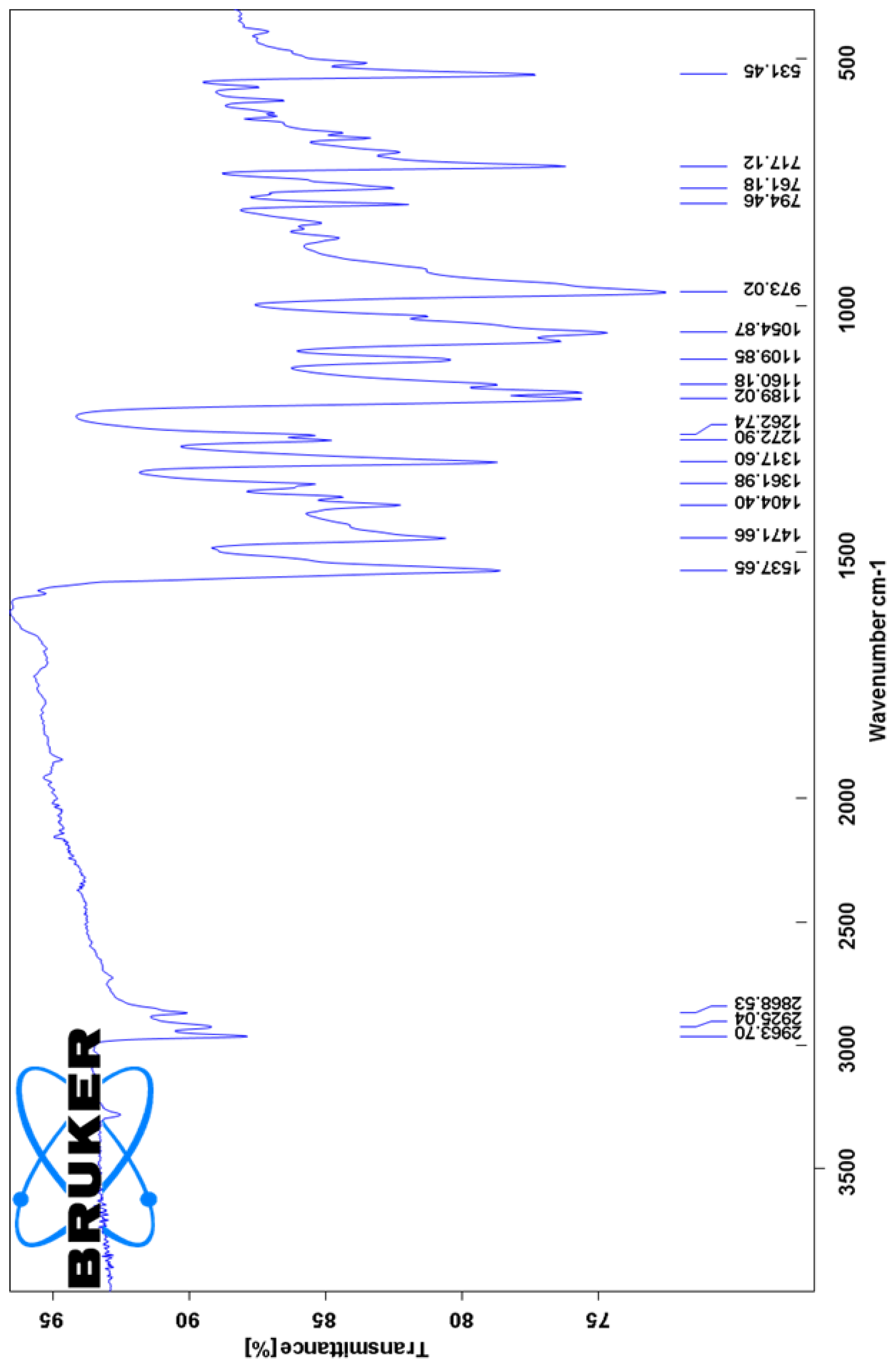

Supplement: Figure S1 — FT-IR spectrum of Compound 1. [file turkjchem-45-6-2024s1.tif]

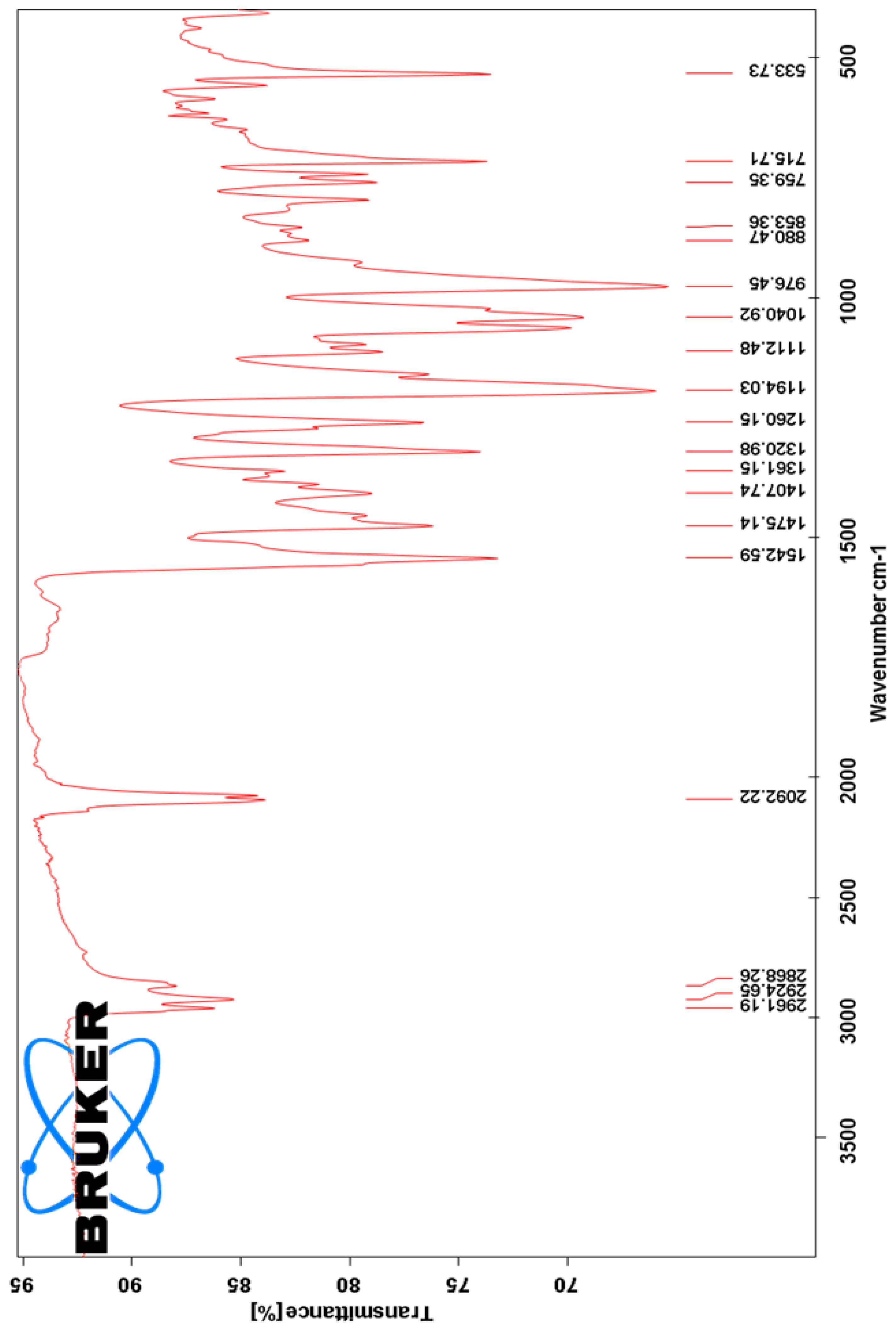

Supplement: Figure S2 — FT-IR spectrum of Compound 2. [file turkjchem-45-6-2024s2.tif]

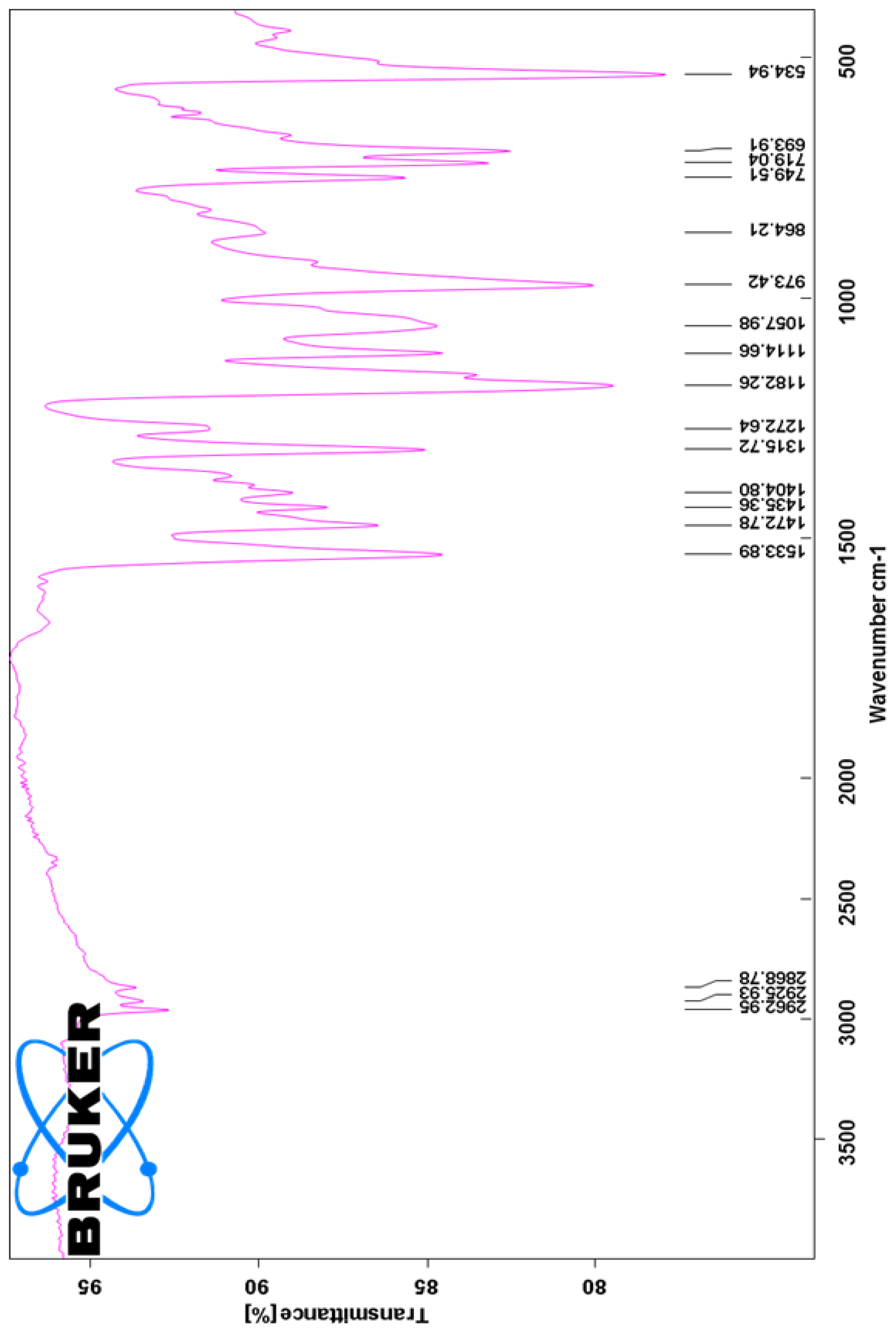

Supplement: Figure S3 — FT-IR spectrum of Compound 3. [file turkjchem-45-6-2024s3.tif]

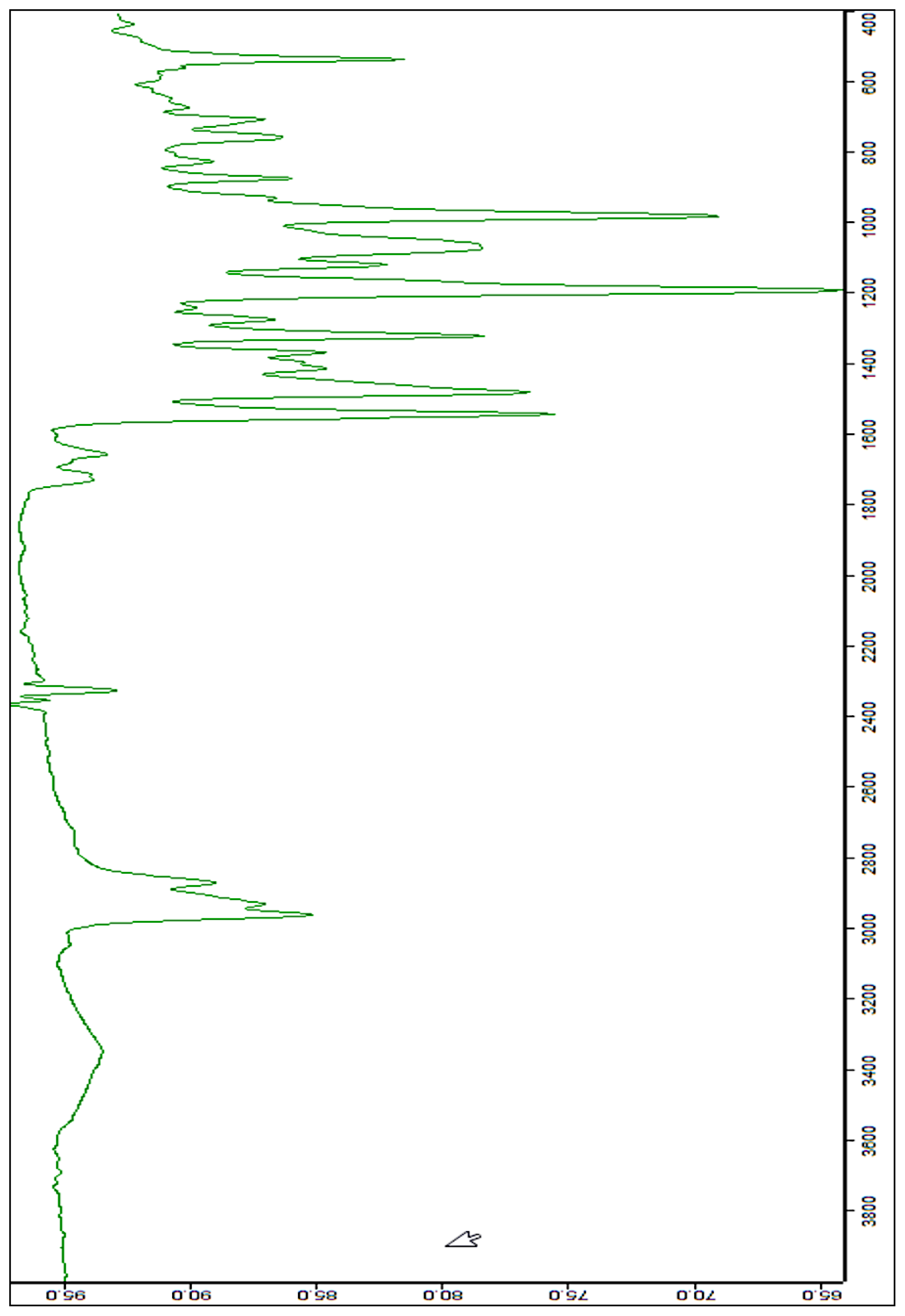

Supplement: Figure S4 — FT-IR spectrum of t-BODIPY. [file turkjchem-45-6-2024s4.tif]

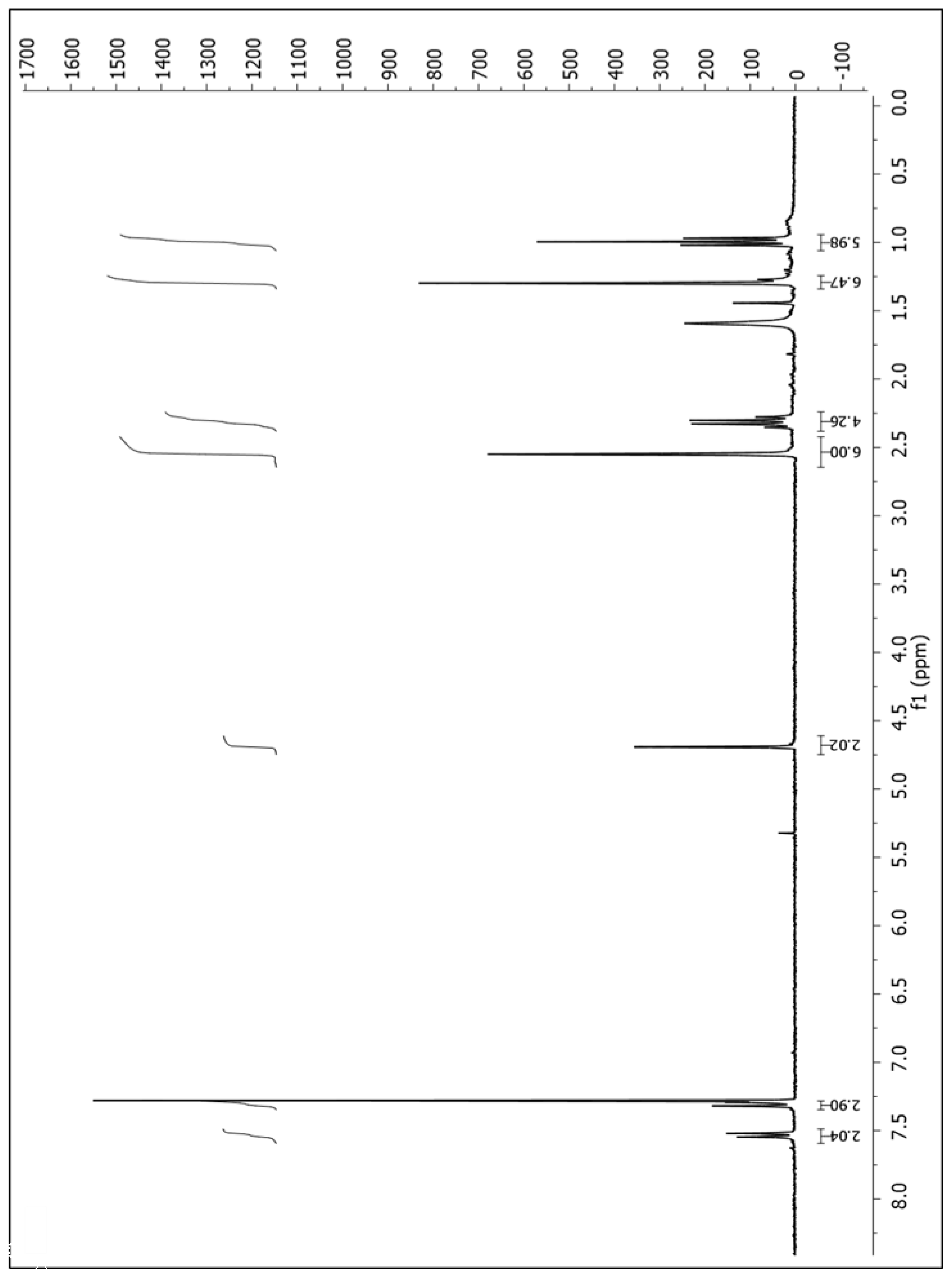

Supplement: Figure S5 — 1H-NMR spectrum of Compound 1. [file turkjchem-45-6-2024s5.tif]

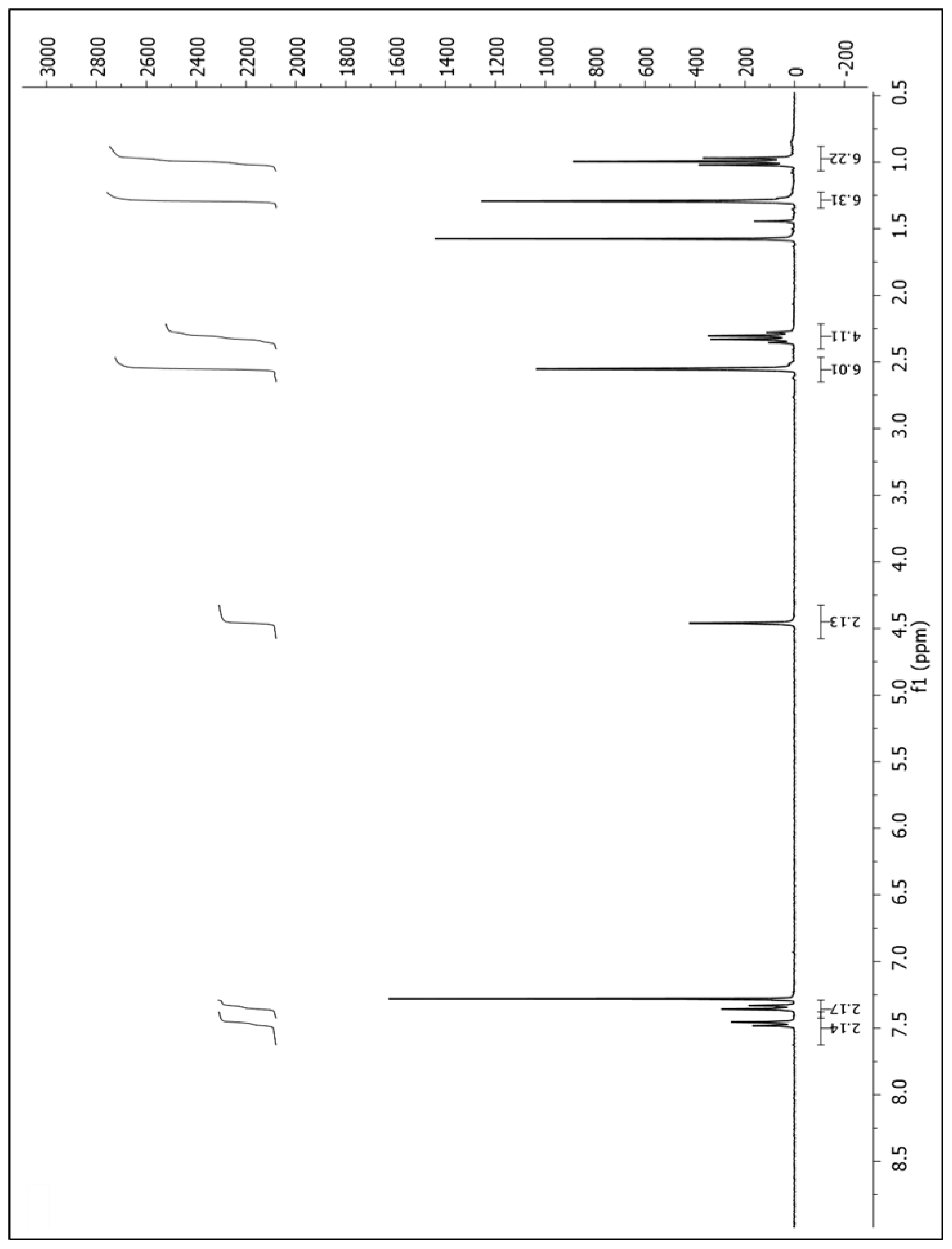

Supplement: Figure S6 — 1H-NMR spectrum of Compound 2 (25 °C). [file turkjchem-45-6-2024s6.tif]

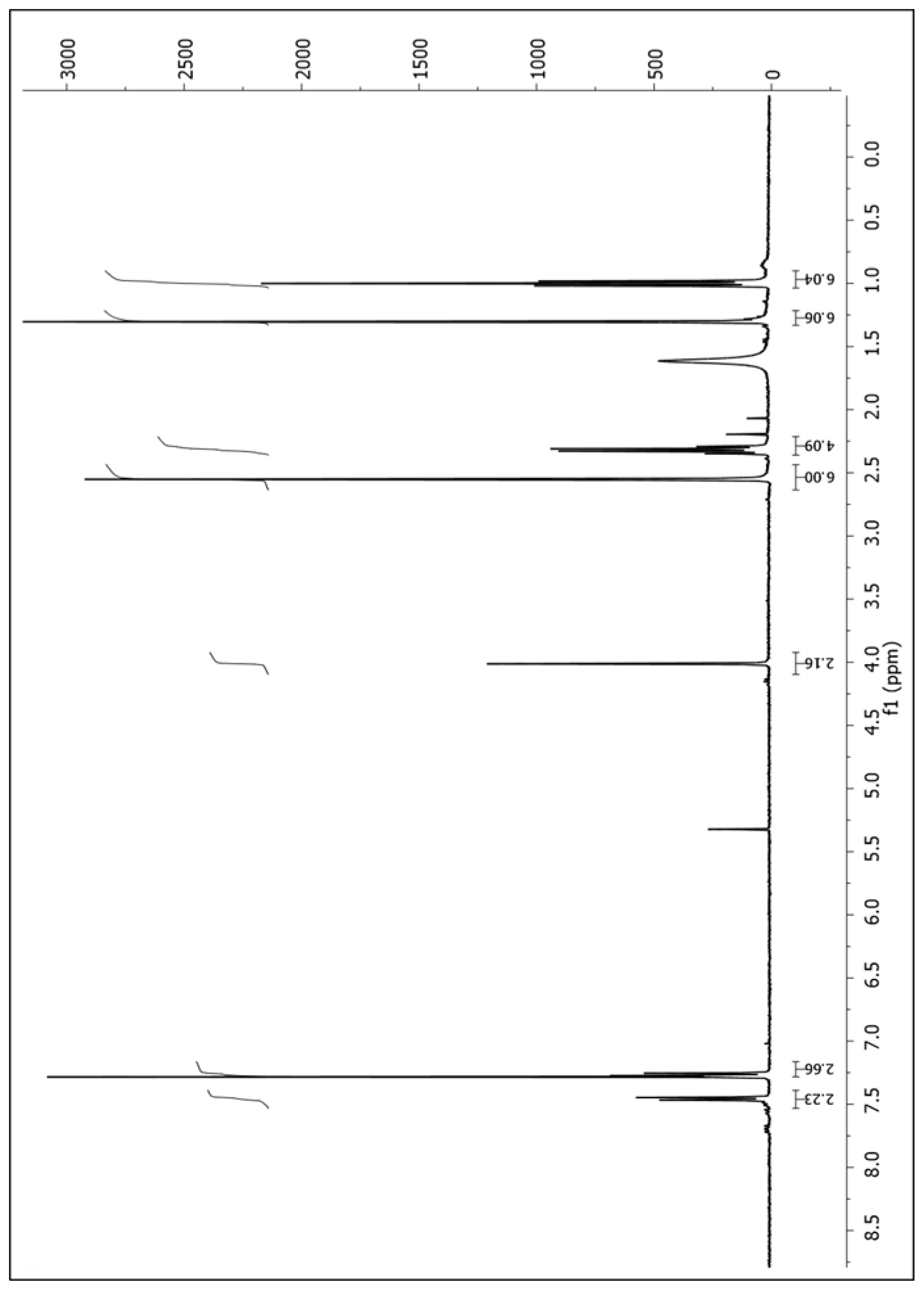

Supplement: Figure S7 — 1H-NMR spectrum of Compound 3 (25 °C). [file turkjchem-45-6-2024s7.tif]

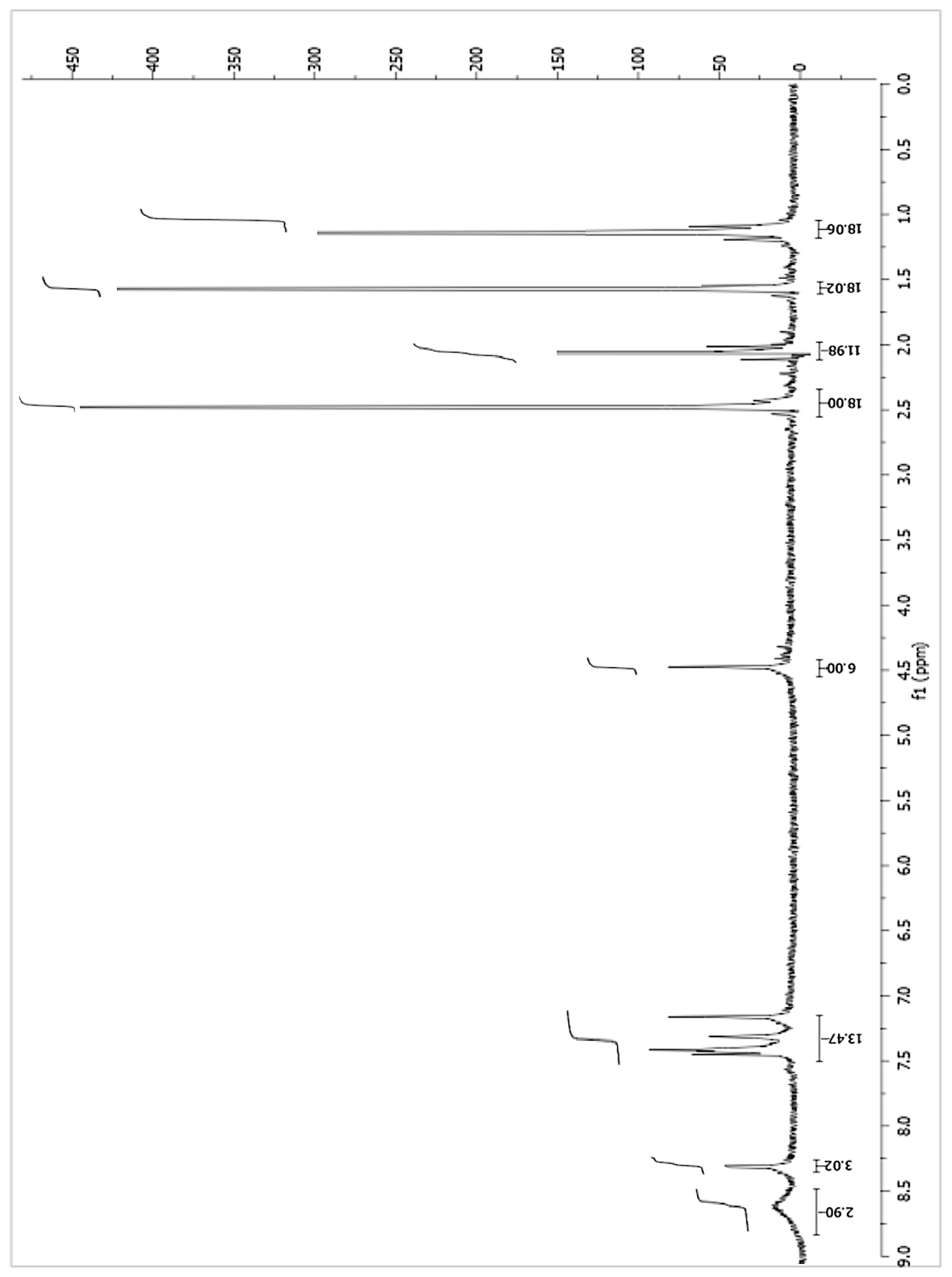

Supplement: Figure S8 — 1H-NMR spectrum of t-BODIPY (25 °C). [file turkjchem-45-6-2024s8.tif]

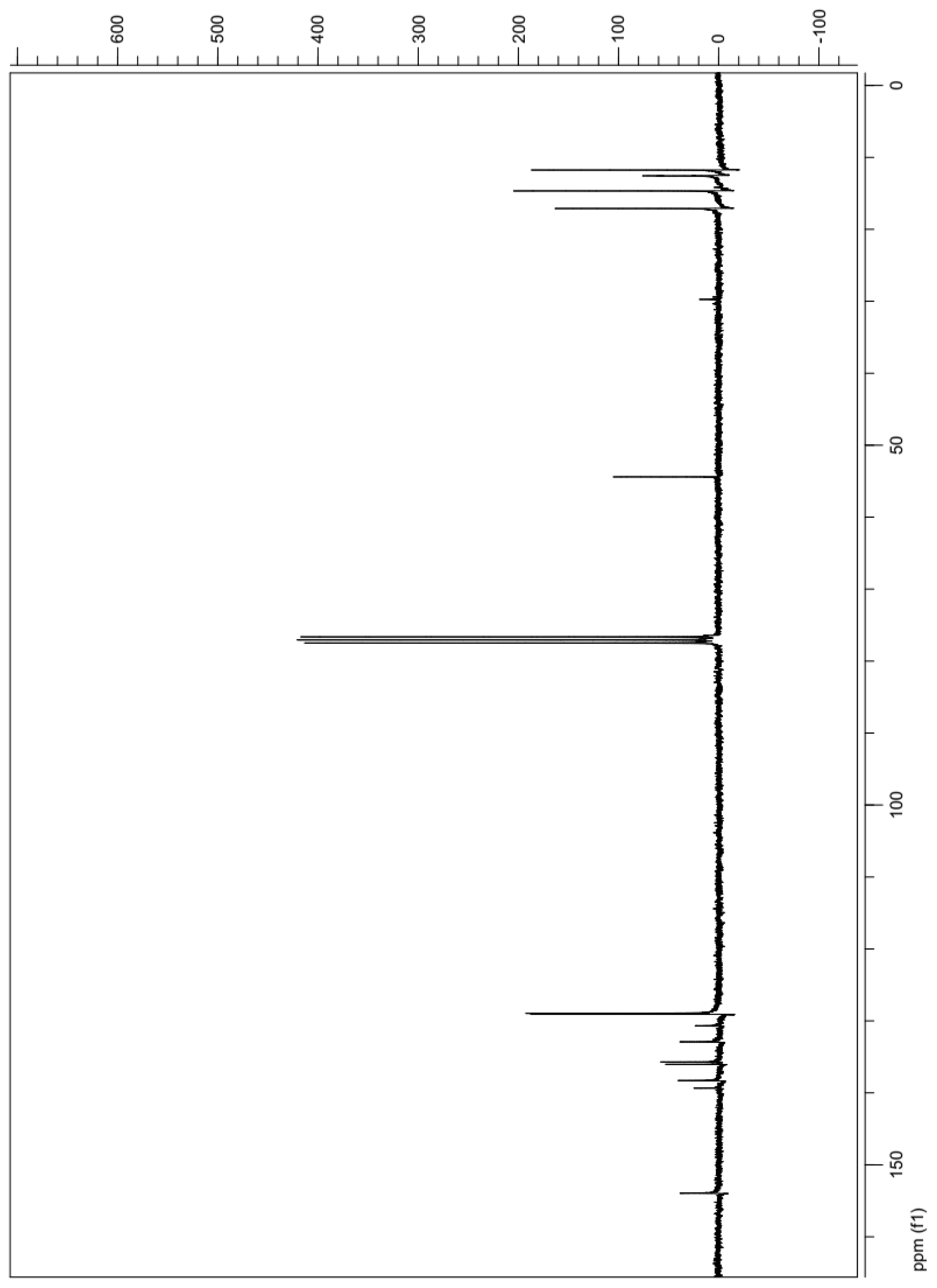

Supplement: Figure S9 — 13C-NMR spectrum of Compound 2 (25 °C). [file turkjchem-45-6-2024s9.tif]

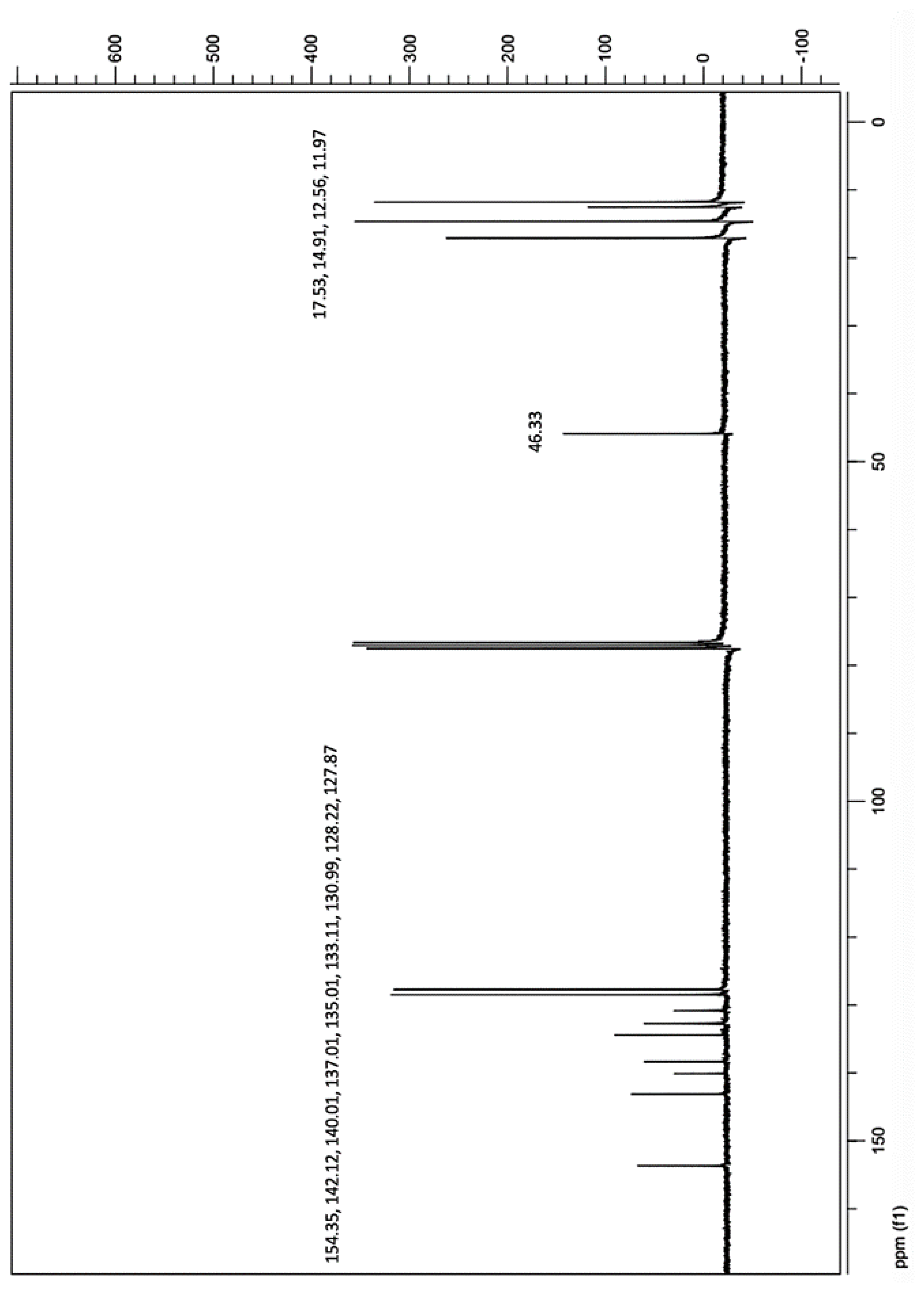

Supplement: Figure S10 — 13C-NMR spectrum of Compound 3 (25 °C). [file turkjchem-45-6-2024s10.tif]

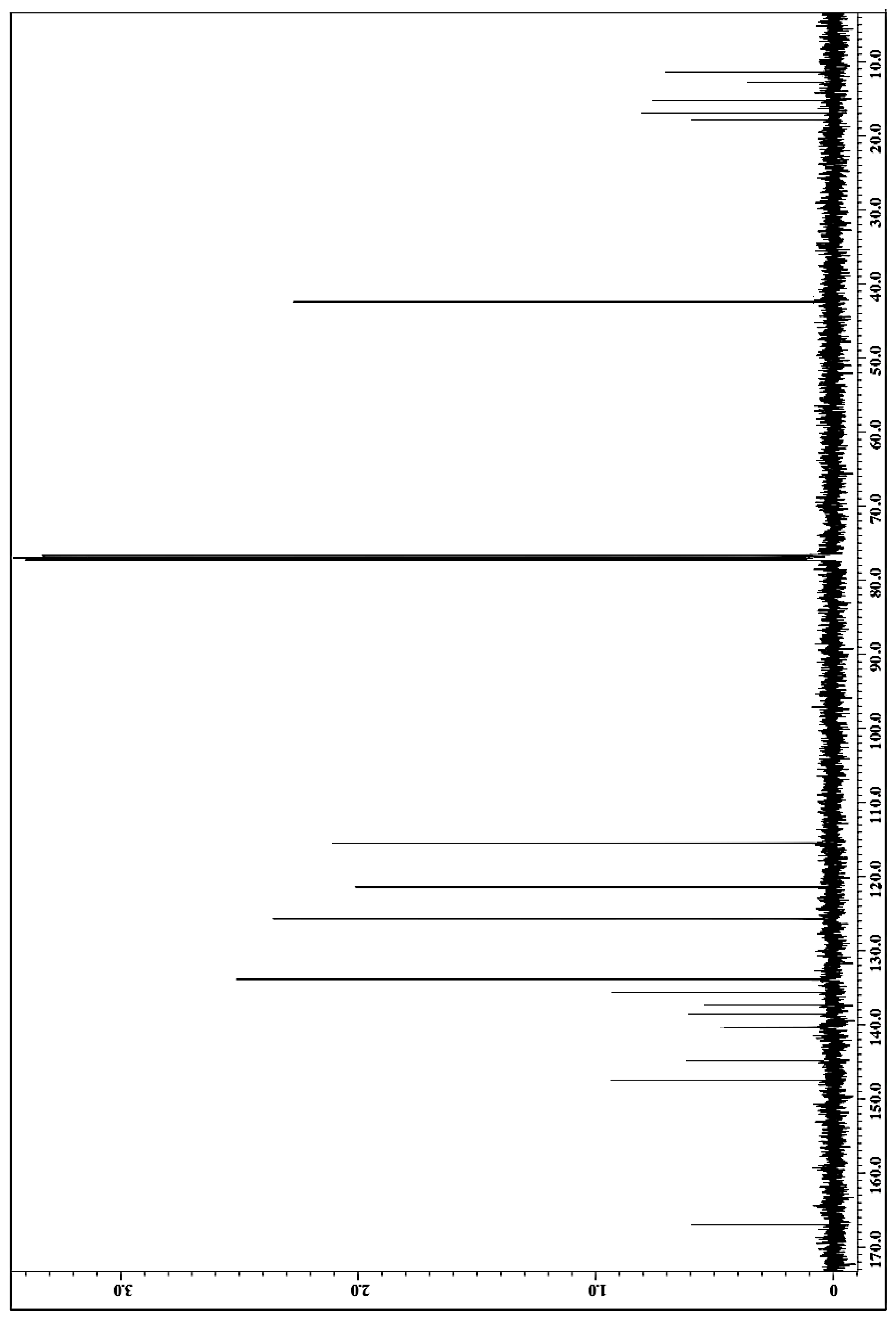

Supplement: Figure S11 — 13C-NMR spectrum of t-BODIPY (25 °C). [file turkjchem-45-6-2024s11.tif]

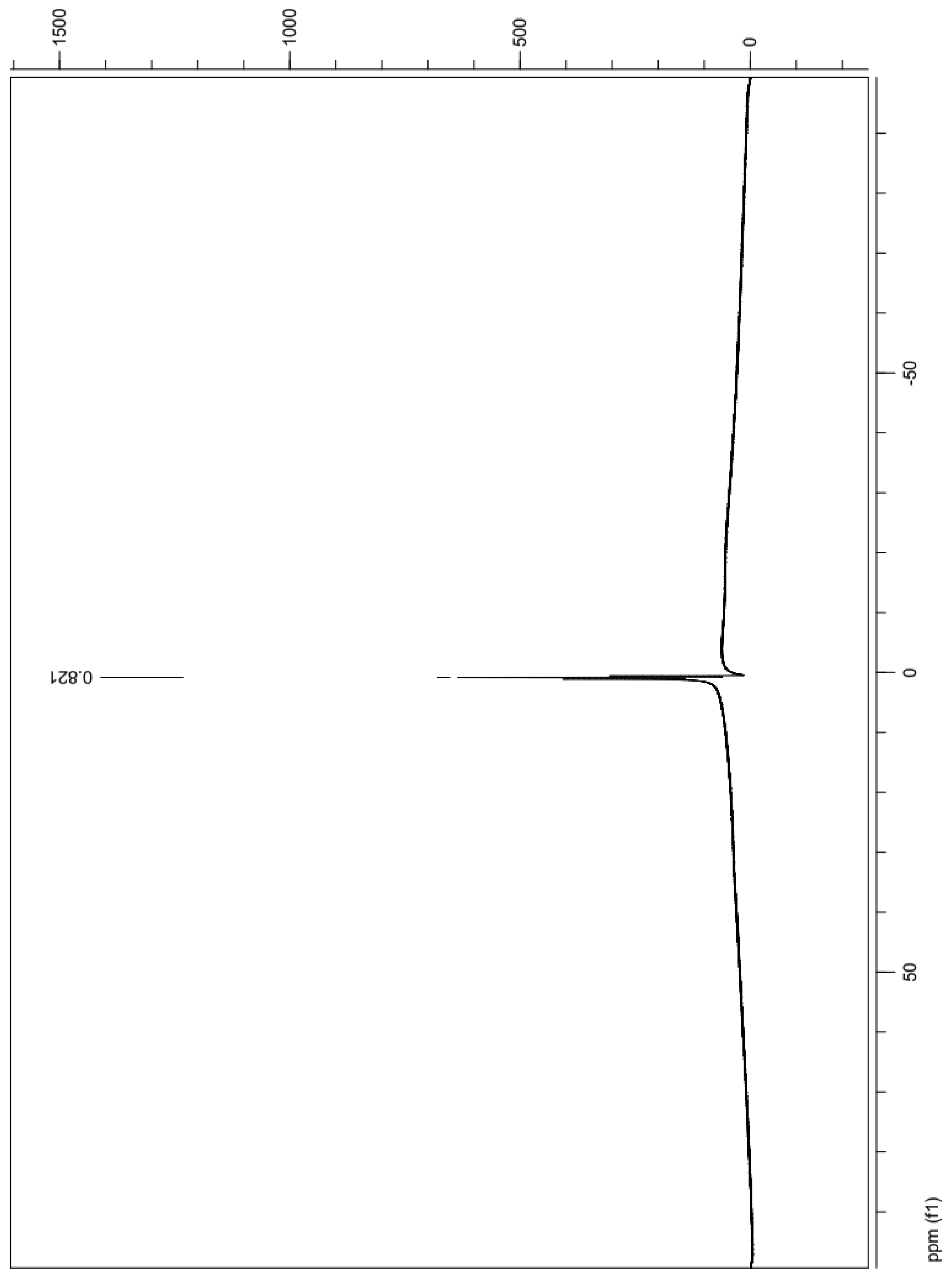

Supplement: Figure S12 — 11B-NMR spectrum of Compound 2 (25 °C). [file turkjchem-45-6-2024s12.tif]

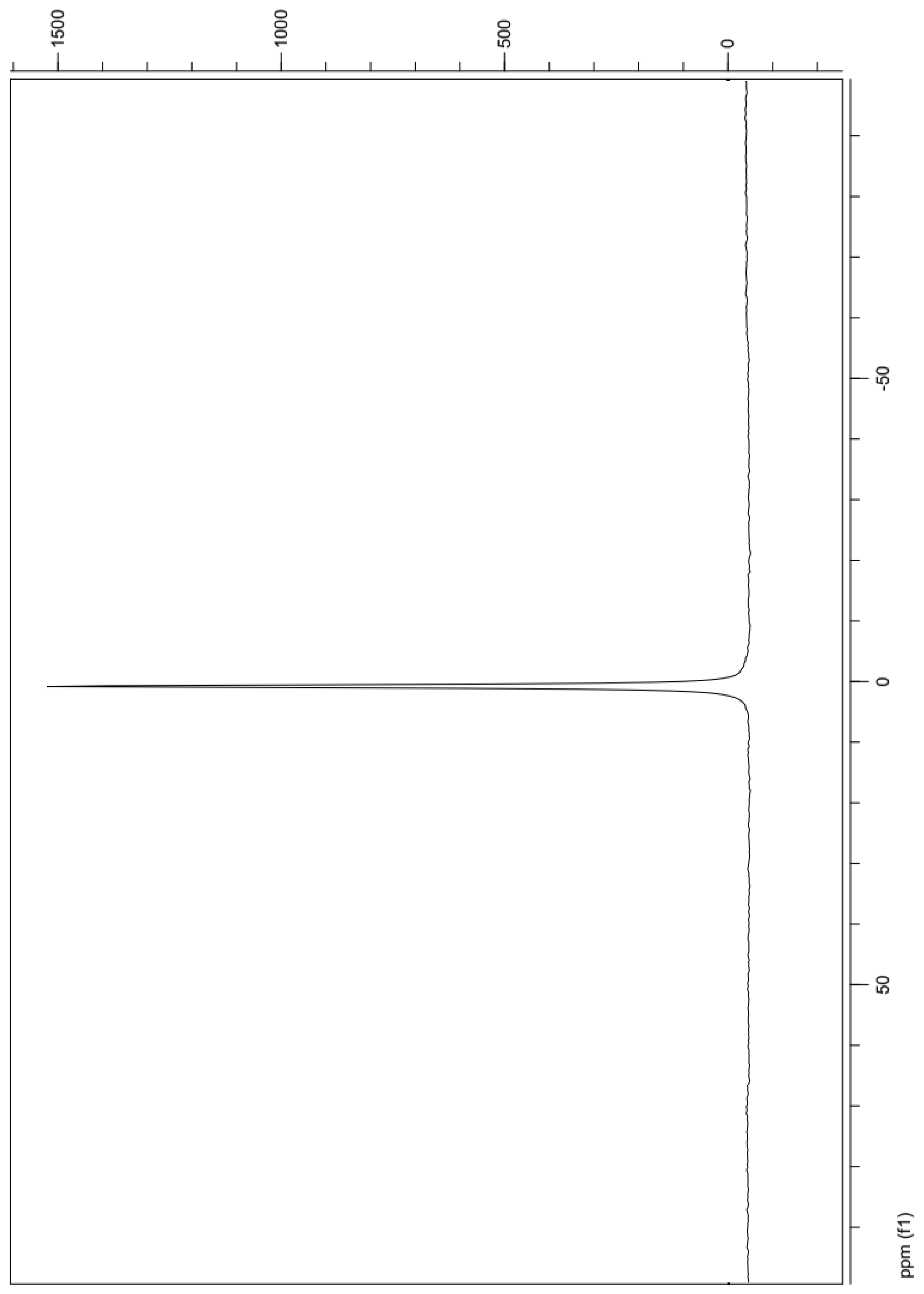

Supplement: Figure S13 — 11B-NMR spectrum of Compound 3 (25 °C). [file turkjchem-45-6-2024s13.tif]

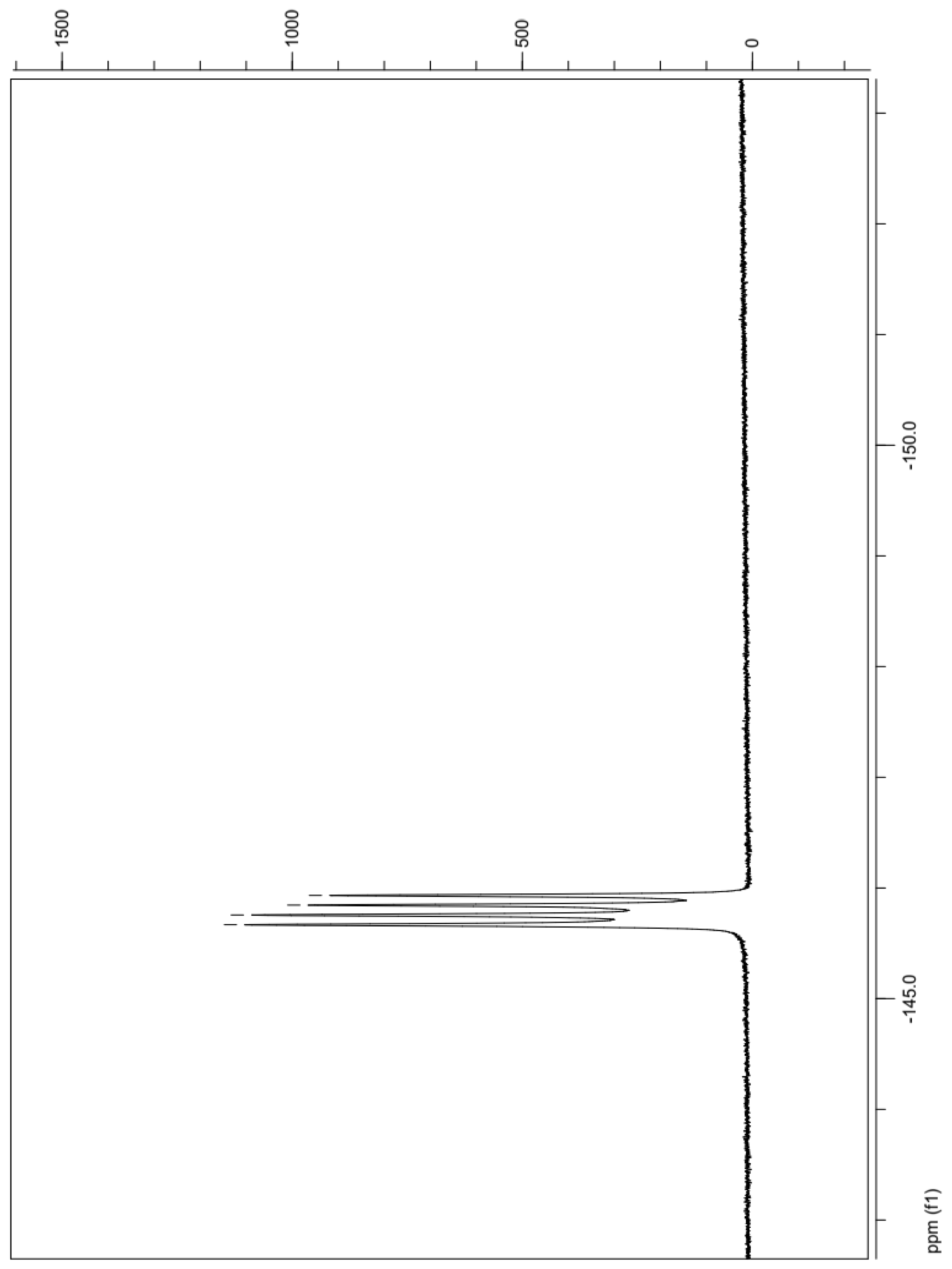

Supplement: Figure S14 — 19F-NMR spectrum of Compound 2 (25 °C). [file turkjchem-45-6-2024s14.tif]

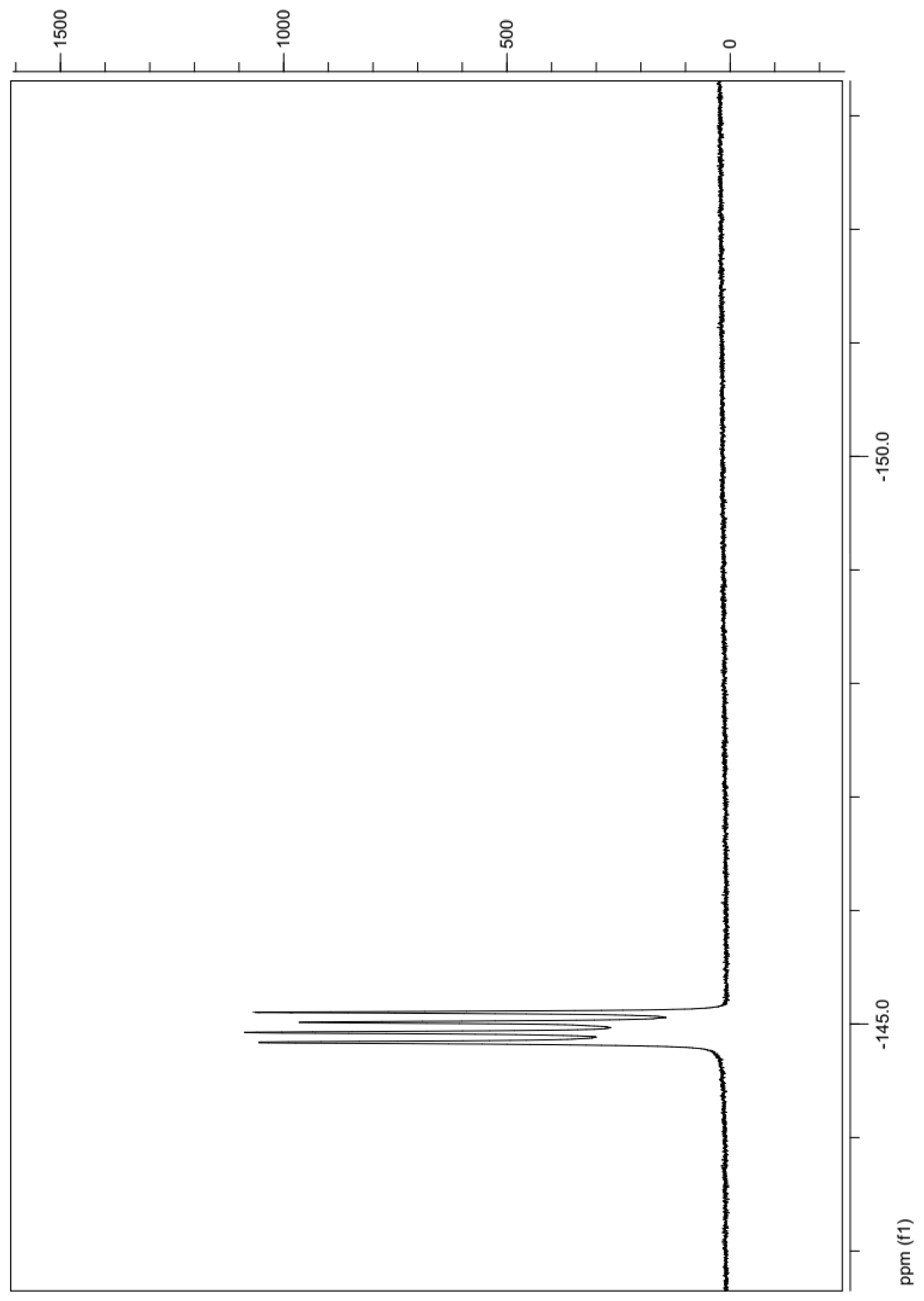

Supplement: Figure S15 — 19F-NMR spectrum of Compound 3 (25 °C). [file turkjchem-45-6-2024s15.tif]

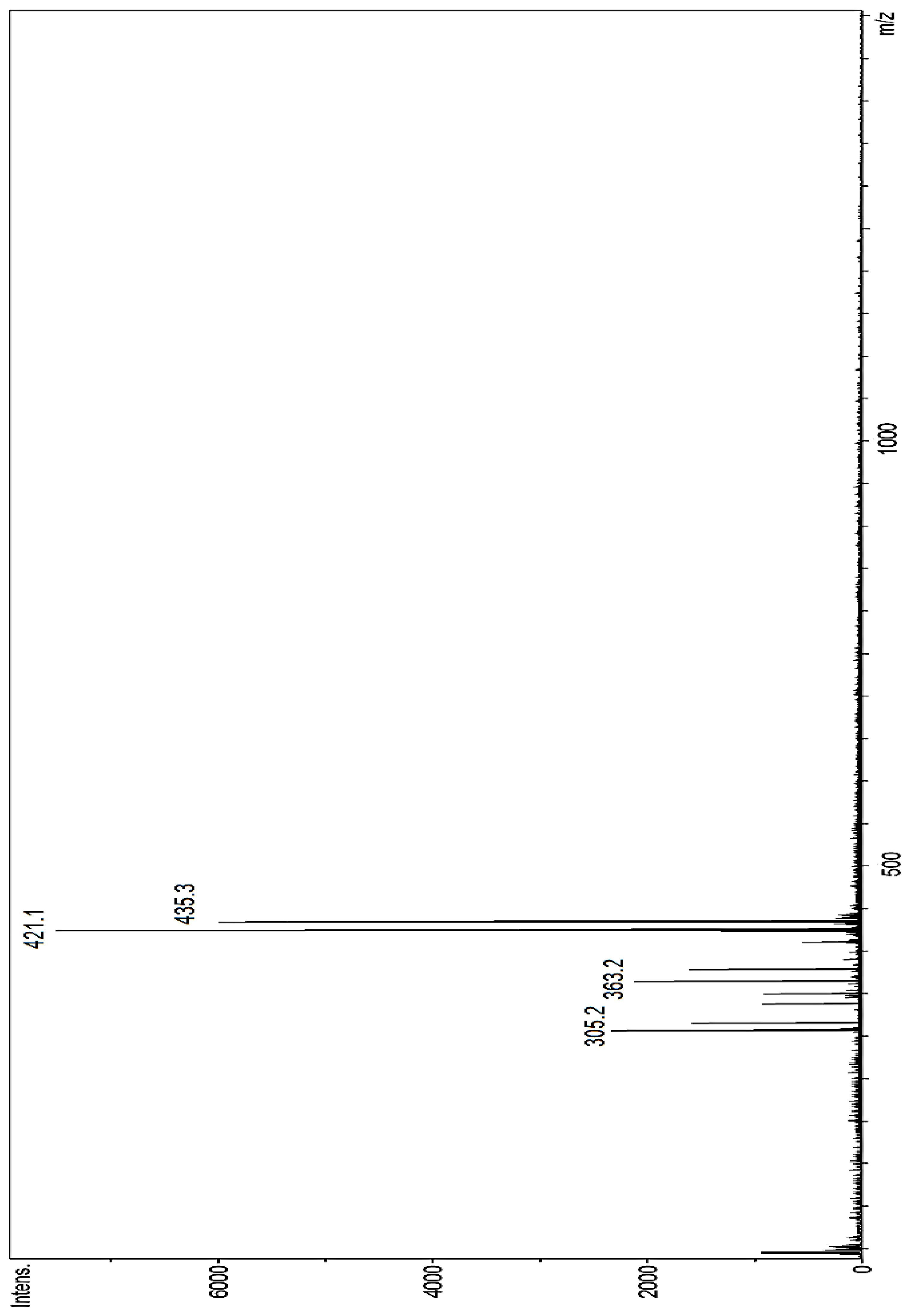

Supplement: Figure S16 — Mass spectrum of Compound 1. [file turkjchem-45-6-2024s16.tif]

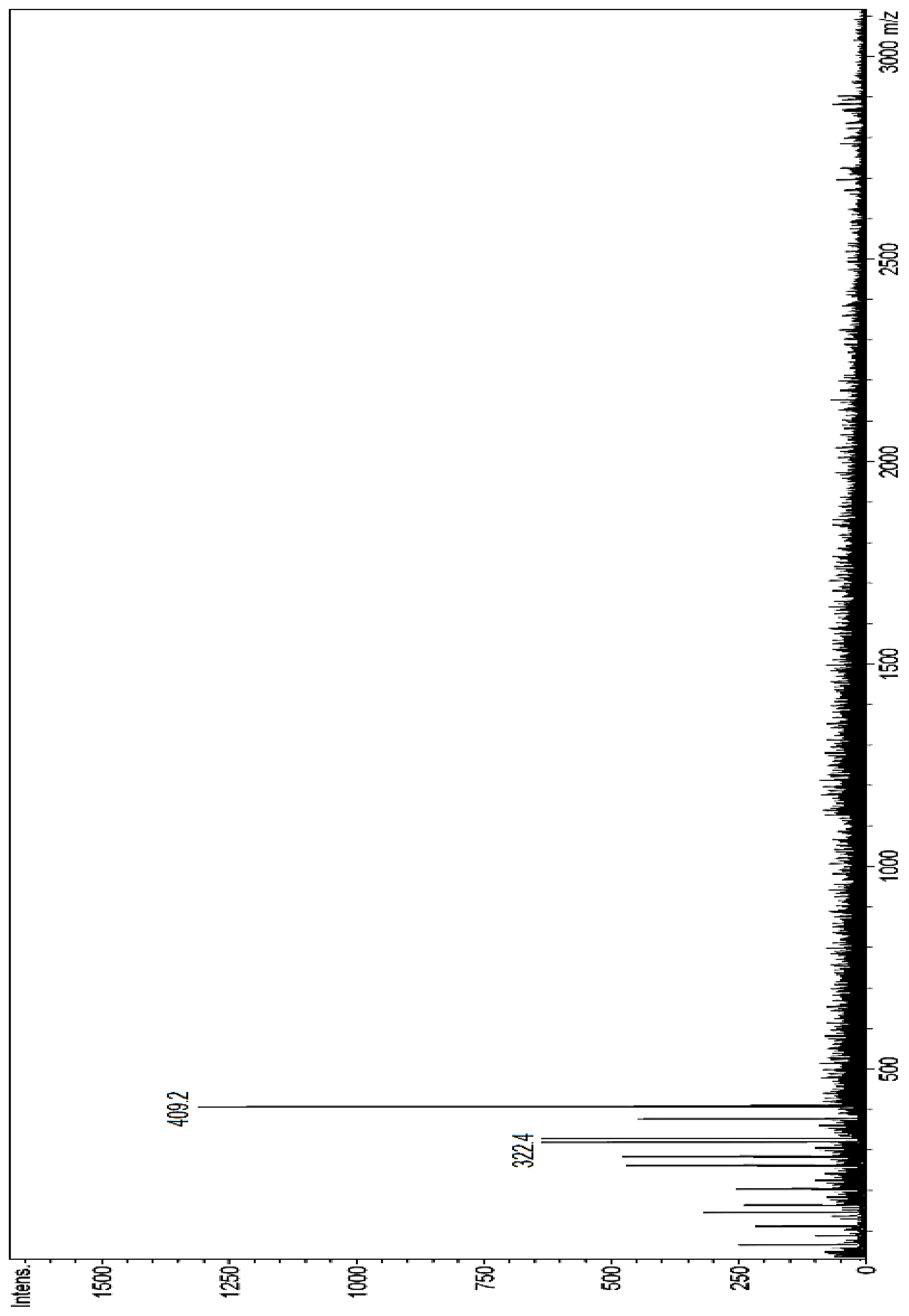

Supplement: Figure S17 — Mass spectrum of Compound 3. [file turkjchem-45-6-2024s17.tif]

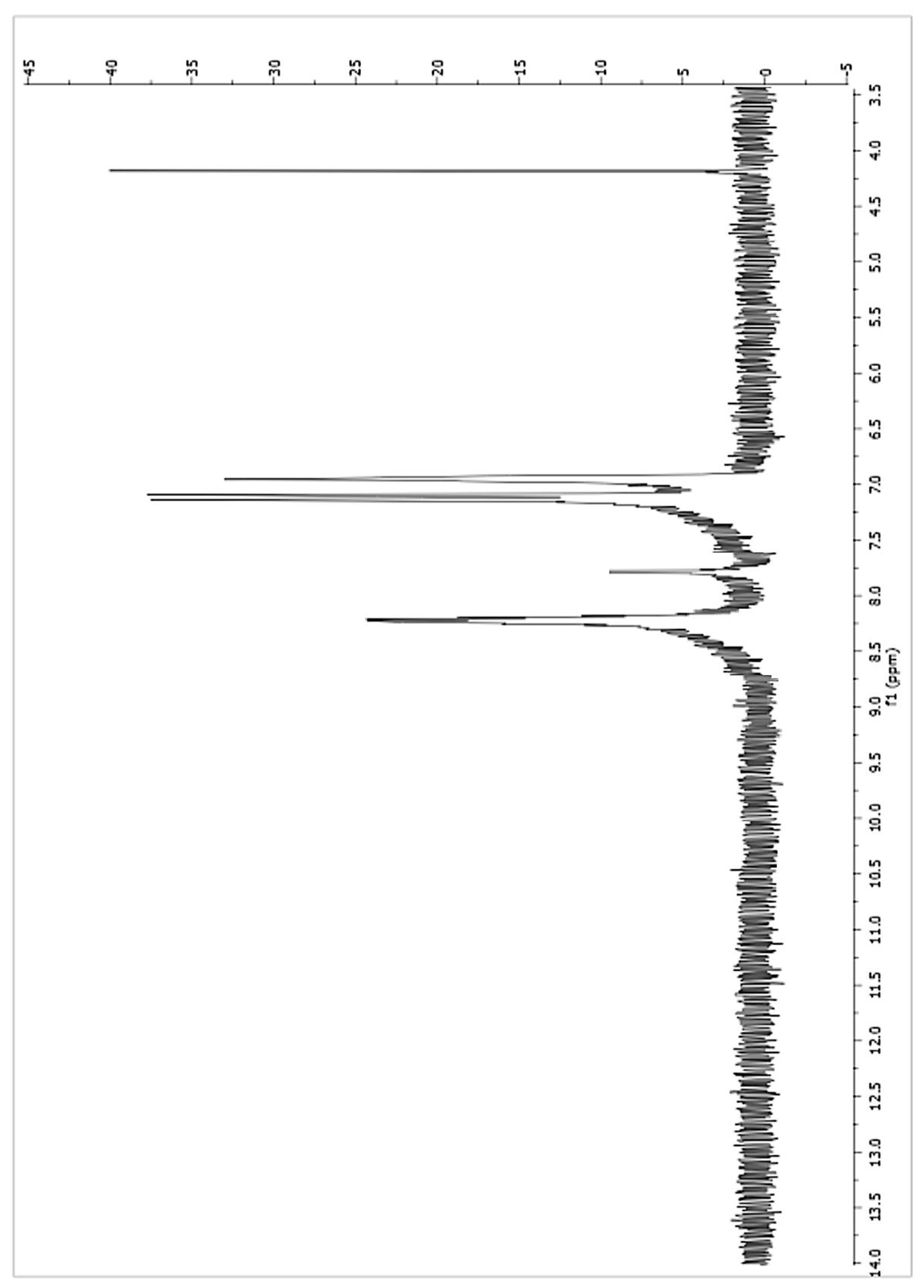

Supplement: Figure S18 — 1H NMR titration spectra of t-BODIPY upon addition of 1 equiv. Cu (II) in DMSO-d6 solution (25 °C). [file turkjchem-45-6-2024s18.tif]

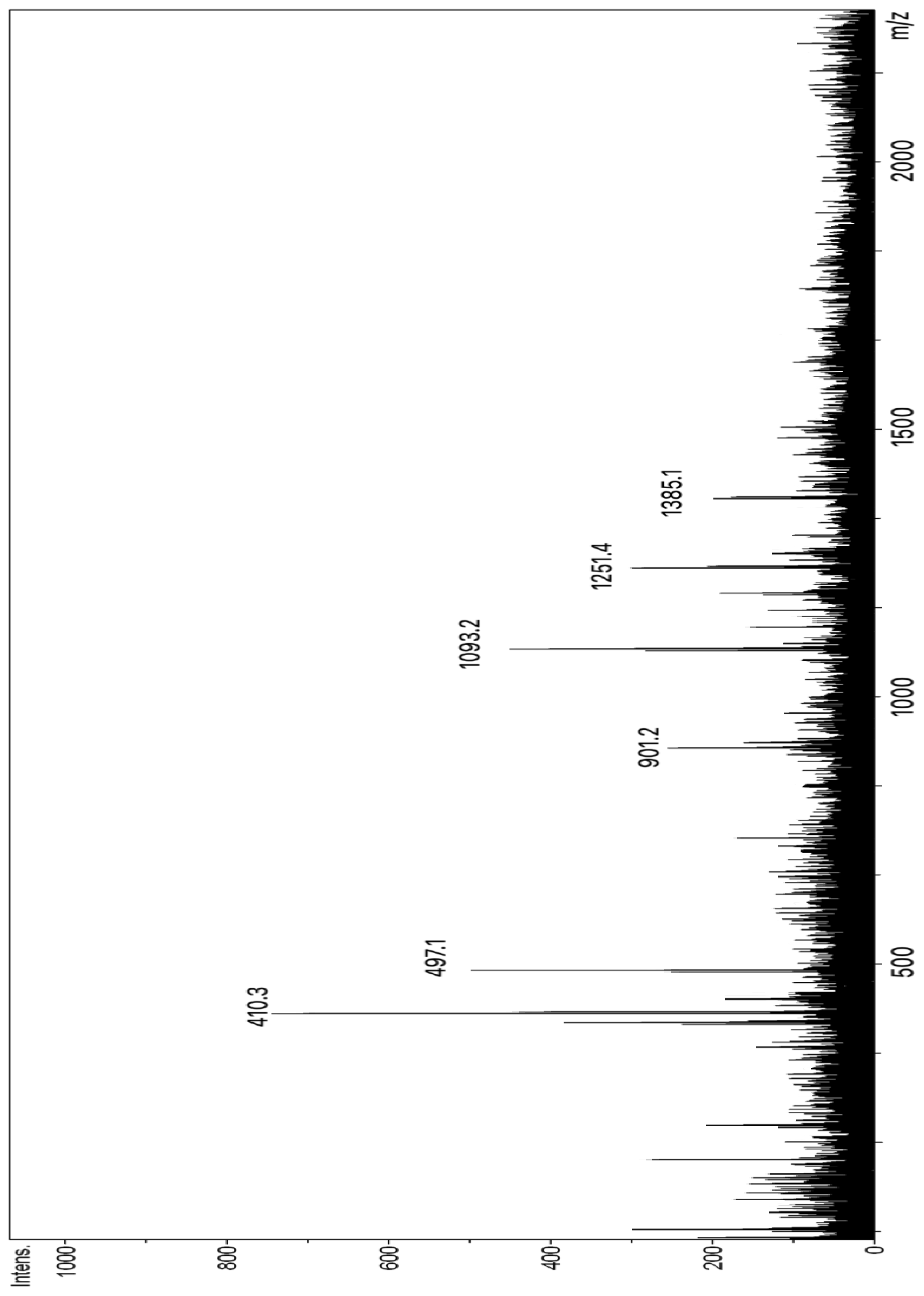

Supplement: Figure S19 — Mass spectrum of t-BODIPY. [file turkjchem-45-6-2024s19.tif]

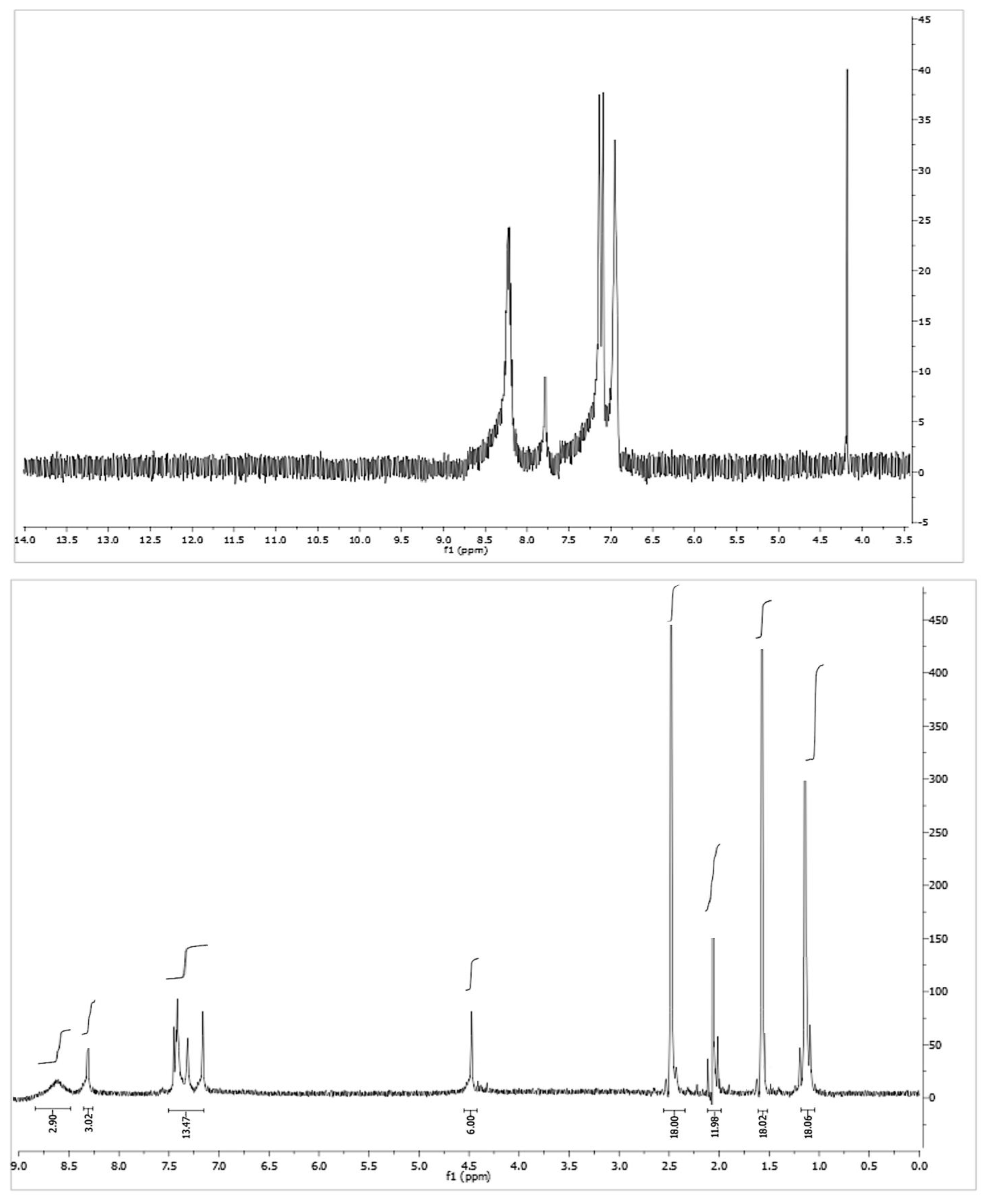

Supplement: Figure S20 — Comparation of 1H NMR titration spectra of t-BODIPY and t-BODIPY-Cu (II) complex. [file turkjchem-45-6-2024s20.tif]

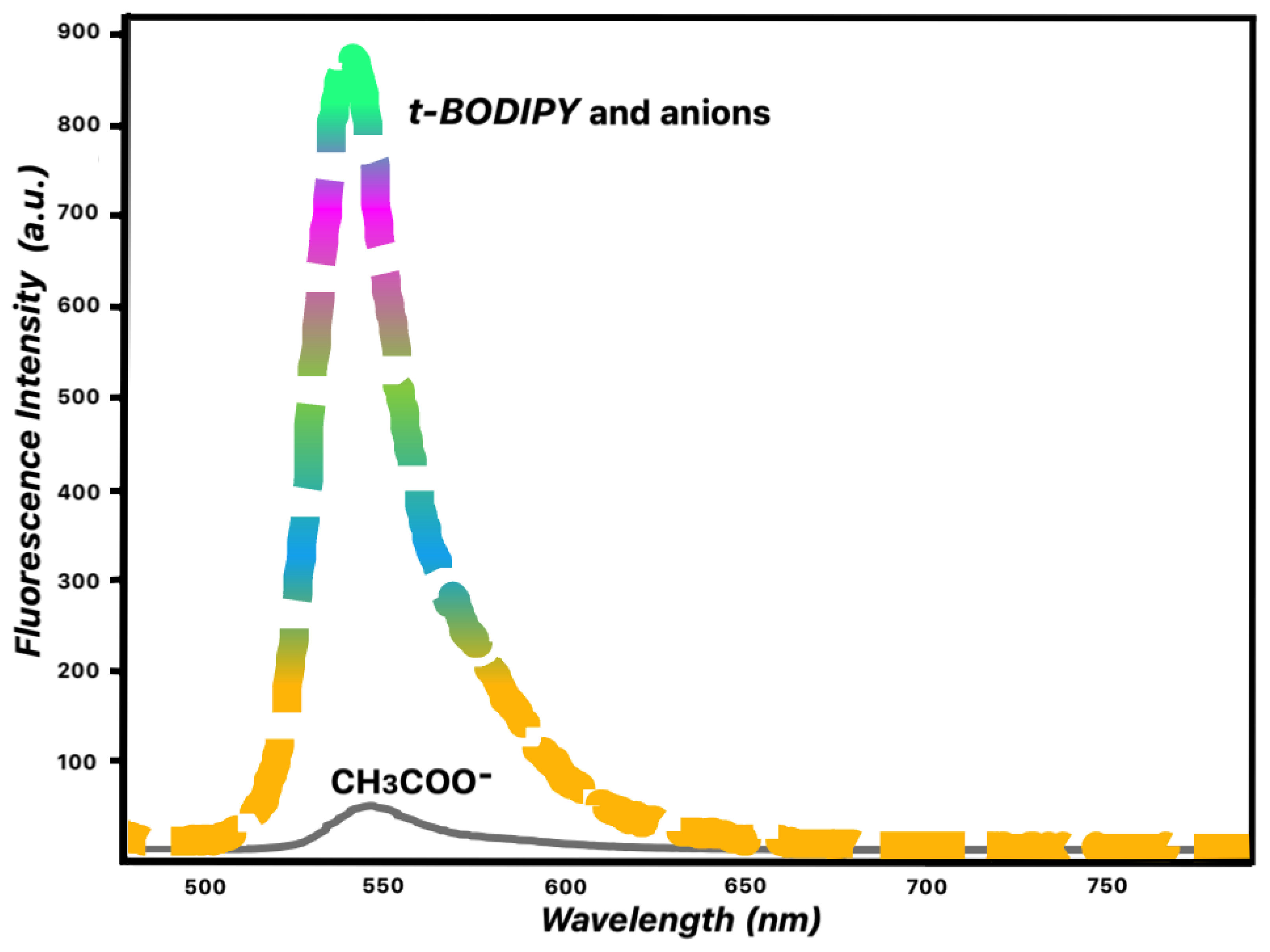

Supplement: Figure S21 — Fluorescence spectral changes of t-BODIPY upon addition of various anions (F−, Cl−, I−, Br−, CH3COO−, HCO3−, CO32−, HSO3−, SO42−, NO3−). λex:470 nm. [file turkjchem-45-6-2024s21.tif]
